# Chronic Morphine Treatment Attenuates Cell Growth of Human BT474 Breast Cancer Cells by Rearrangement of the ErbB Signalling Network

**DOI:** 10.1371/journal.pone.0053510

**Published:** 2013-01-07

**Authors:** Inka Regine Weingaertner, Sarah Koutnik, Hermann Ammer

**Affiliations:** Institute of Pharmacology, Toxicology and Pharmacy, Ludwig-Maximilians-University, Munich, Germany; Wayne State University School of Medicine, United States of America

## Abstract

**Background:**

There is increasing evidence that opioid analgesics may interfere with tumour growth. It is currently thought that these effects are mediated by transactivation of receptor tyrosine kinase (RTK)-controlled ERK1/2 and Akt signalling. The growth of many breast cancer cells is dependent on hyperactive ErbB receptor networks and one of the most successful approaches in antineoplastic therapy during the last decade was the development of ErbB-targeted therapies. However, the response rates of single therapies are often poor and resistance mechanisms evolve rapidly. To date there is no information about the ability of opioid analgesics to interfere with the growth of ErbB-driven cancers.

**Methods and Principal Findings:**

Here we demonstrate that ErbB2 overexpressing BT474 human breast cancer cells carry fully functional endogenous µ-opioid receptors. Most interestingly, the acute opioid effects on basal and Heregulin-stimulated ERK1/2 and Akt phosphorylation changed considerably during chronic Morphine treatment. Investigation of the underlying mechanism by the use of protein kinase inhibitors and co-immunoprecipitation studies revealed that chronic Morphine treatment results in rearrangement of the ErbB signalling network leading to dissociation of ERK1/2 from Akt signalling and a switch from ErbB1/ErbB3 to ErbB1/ErbB2-dependent cell growth. In chronically Morphine-treated cells Heregulin-stimulated ERK1/2 signalling is redirected via a newly established PI3K- and metalloproteinase-dependent feedback loop. Together, these alterations result in apoptosis of BT474 cells. A similar switch in Heregulin-stimulated ERK1/2 signalling from an ErbB2-independent to an ErbB2-, PI3K- and metalloproteinase-dependent mechanism was also observed in κ-opioid receptor expressing SKBR3 human mammary adenocarcinoma cells.

**Conclusions and Significance:**

The present data demonstrate that the ErbB receptor network of human breast cancer cells represents a target for chronic Morphine treatment. Rearrangement of ErbB signalling by chronic Morphine may provide a promising strategy to enhance the sensitivity of breast cancer cells to ErbB-directed therapies and to prevent the development of escape mechanisms.

## Introduction

Opioids are potent analgesics and widely used for anaesthetic pre-medication and management of cancer pain. They mediate their action via specific binding sites (δ, κ, µ) that belong to the family of G protein-coupled receptors. Opioid receptors are predominantly expressed in neuronal tissues and inhibit neuronal excitability by regulating their “classical” effector systems adenylyl cyclase, potassium channels and voltage-dependent calcium currents [1]. Beside this, opioid receptors may also regulate the activity of a variety of mitogen-activated protein (MAP) kinases, including Extracellular Signal-Regulated Kinases 1 and 2 (ERK1/2), c-Jun N-terminal Kinase (JNK), p38, Signal Transducer and Activator of Transcription 5 (STAT5) and Protein Kinase B (PKB/Akt) [2], [3]. Activation of these “non classical” opioid effector systems is mediated via transactivation of receptor tyrosine kinase (RTK)-associated ERK1/2 and Akt signalling pathways [4], [5]. Due to the ability of opioid receptors to regulate the dominant RTK system in a given cellular context [6], chronic opioid treatment might provide a means to selectively interfere with tumour cell growth. Because the opioid effects on tumour cell proliferation and apoptosis reported so far are rather discrepant and role of opioid receptors in these studies was not always clear [7], [8], the aim of the present study was to investigate chronic Morphine regulation of RTK-dependent cell growth in a defined tumour cell model carrying endogenous µ-opioid receptors.

The human Epidermal Growth Factor (EGF) Receptor family (ErbB, also termed HER) consists of four members (ErbB1-4) and belongs to subclass I of the superfamily of RTKs. They are activated by more than 10 different growth factor ligands with partly overlapping (EGF, HB-EGF, TGF-α, and Betacellulin) or more discrete (Neuregulins) receptor specificities [9]. ErbB receptors are transmembrane receptors consisting of an extracellular ligand binding domain, an intracellular kinase domain and an intracellular C-terminal tail. Ligand binding favours receptor dimerization, which in turn leads to activation of the intracellular kinase domain and autophosphorylation of distinct tyrosine residues in the C-terminal tail. These provide docking sites for binding of the Shc/Grb2/SOS complex linking ErbB receptors to activation of the mitogenic Ras/Raf/ERK1/2 signalling module [10]. Although structurally highly homologous, individual ErbB receptors differ with respect to ligand binding and kinase activity. Most importantly, there is currently no endogenous ligand known for ErbB2 [11], whereas ErbB3 lacks catalytic tyrosine kinase activity [12]. Thus, both receptors must undergo heterodimerization for signalling. While ErbB2 is considered a signal amplifier, activated ErbB3 signal through their dimerization partner. In ErbB1/ErbB3 heterodimers, ligand activation of ErbB3 results in ErbB1-mediated stimulation of the Ras/Raf/ERK1/2 pathway. While all ErbB family members are able to cross-regulate the anti-apoptotic Phosphatidylinositol 3-kinase (PI3K)/Akt pathway in a Ras-dependent manner, ErbB3 may also directly activate all 3 regulatory subunits of PI3K in the presence of ligand activated binding partners [13]. Due to the ability of ErbB receptors to form multiple homo- and heterodimers that considerably differ in their signalling capacity, alterations in receptor abundance and dimerization will have dramatic consequences on mitogenic and anti-apoptotic signalling [9].

Human BT474 breast cancer cells were originally isolated from a solid, invasive ductal carcinoma of the breast from a 60 years old woman [14]. These cells are characterized by a dysregulated ErbB receptor system, because they overexpress the ErbB2 receptor [11]. ErbB2 is present in about 25–30% of breast cancer patients [15] and is associated with poor prognosis and high relapse rate [11]. BT474 cells also carry physiologic levels of ErbB1 and ErbB3 and low levels of ErbB4 [16], providing them a suitable model system for investigating the impact of ErbB signalling on tumour cell growth and ErbB2-targeted anti-tumour strategies [17]. In the present study we demonstrate that BT474 cells carry endogenous µ-opioid receptors and that chronic Morphine treatment attenuates Heregulin-induced cell growth and migration. The underlying mechanism involves dissociation of Heregulin-induced ERK1/2 and Akt signalling from a common ErbB1/ErbB3 to separate ErbB1/ErbB2 and ErbB1/ErbB3 mechanisms resulting in PI3K-dependent cell growth. Similar chronic Morphine effects on ErbB signalling were also observed in ErbB2 overexpressing SKBR3 human mammary adenocarcinoma cells that carry endogenous κ-opioid receptors. These results demonstrate that chronic Morphine treatment is able to interfere with the growth characteristics of opioid receptor expressing tumour cells by rearrangement of the ErbB receptor system. Such a mechanism might provide a promising strategy to enhance the sensitivity of ErbB-directed antineoplastic strategies and to prevent escape mechanisms.

## Materials and Methods

### Materials

Foetal calf serum and cell culture reagents were from Pan Biotech. Forskolin, human recombinant insulin, 3-isobutyl-1-methylxanthin (IBMX), (-)-Naloxone-HCl, and all standard laboratory reagents were from Sigma-Aldrich. (-)-Morphine-HCl was from Merck, Heregulin (C-terminal fragment of Heregulin β1) from R&D Systems, recombinant epidermal growth factor (EGF) from Bachem and [^3^H]DAMGO (30–60 Ci/mmol) from PerkinElmer. Tyrphostin AG1478, AG825, Epigallocatechingallat (EGCG) and Wortmannin, were from Calbiochem. Protein A-agarose beads and dithiobis-(succinimidyl propionate) (DSP) were purchased from Thermo Scientific. Anti-G protein antibodies have been produced in our laboratory (G_i_α2, G_i_α3, G_s_α, Gβ) or were obtained from Gramsch Laboratories (G_q/11_α, G_12_α, G_13_α, G_14_α). Primary antibodies recognizing ERK1/2, phospho-ERK1/2 [Thr202/Tyr204], Akt, phospho-Akt [Ser473], ErbB1, ErbB2, ErbB3, ErbB4, β-tubulin as well as cleaved PARP [Asp214] were from Cell Signalling Technology. Secondary horseradish peroxidase-conjugated anti-mouse and anti-rabbit IgG were from Promega and Cell Signalling.

### Cell Culture, Chronic Opioid Treatment and Membrane Preparation

BT474 cells were purchased from Cell Lines Service Inc. and grown in RPMI 1640 medium supplemented with 10% heat-inactivated foetal calf serum (FCS), 100 IU/ml Penicillin, 0.1 mg/ml Streptomycin, 0.2% Enrofloxacin and 0.02 mg/ml Insulin at 37°C in a humidified atmosphere of 5% CO2 in air. SKBR3 cells were obtained from American Type Culture Collection (ATCC) and grown in McCoy's 5A modified medium plus supplements as above. For experimentation, BT474 cells were seeded onto 96-, 24-, 12- and 6 well dishes as indicated at a density of 0.25×104–5×105 cells per well, left to adhere overnight and then subjected to chronic opioid treatment by the addition of Morphine (10 µM) to the culture medium for 5 d with medium changes and drug renewal every other day.

For evaluation of membrane proteins (µ-opioid and ErbB receptors, G proteins) and radioligand binding studies, cells were grown in 75 or 150 cm2 culture flasks and plasma membranes were prepared as described [18]. In brief, cells were washed 3 times with ice-cold phosphate-buffered saline (PBS; pH7.4), scraped into 20 ml of TED buffer (5 mM Tris-HCl, 1 mM EDTA, and 1 mM DTT; pH 7.4) and then homogenized for 10 sec using an Ultra Turrax. After removal of unbroken cells (300×g; 10 min), membranes were recovered from the supernatant fraction by centrifugation for 30 min at 20,000×g. The membrane pellet was washed once as above, resuspended in TM buffer (50 mM Tris-HCl, 5 mM MgCl2; pH 7.4) and stored in aliquots at −80°C until use.

### RT-PCR

Total RNA was isolated using Trifast™ reagent (Peqlab) and transcribed into cDNA using the Fist Strand cDNA Synthesis Kit® (Fermentas) according to manufactureŕs protocols. Integrity of cDNA was controlled by determination of GAPDH. Opioid receptors (OR) and adenylyl cyclase isoforms (AC) were amplified using specific primer pairs given in Table 1. All PCR reactions (50 µl) contained 10 pmol of each primer, 10 ng of cDNA, 0.2 mM dNTPs, 1.25 U Pfu DNA polymerase and 20 mM MgSO_4_. Amplified fragments (20 µl) were resolved on a 1.5% agarose gel, stained with ethidium bromide and visualized under UV light.

**Table 1:** Primers and reaction conditions used for amplification of adenylyl cyclases, opioid receptors and GAPDH in human breast cancer cells.

Target	Forward Primer	Reverse Primer	Temp.	Cyc.
AC1	5′-CCGAGTTGGCATCAATGTTGGC	5′-TCCTCAGTCACCTGGATTCTGC	56°C	35
**AC2**	5′-CAGCATCTCTTCAGACCTCGC	5′-CCGGAATGGAGGCAAACATG	57°C	30
**AC3**	5′-GAAGACAAGTCCGAGAGAGAGC	5′-TCTTCTACCACCTGAATGTTGC	57°C	30
**AC4**	5′-TCTCTCCAACTTCATCATCC	5′-ACACTGATGAGAGGCAGAGACC	56°C	35
**AC5**	5′-ACAGGAGCACAACATCAGCG	5′-TGAAGAAGTCTATGGCGTTGGC	58°C	30
**AC6**	5′-TATGACCTACTGCTTGGCGTCC	5′-TAGTAGAGTTCATCATTGCGGC	57°C	30
**AC7**	5′-TGGTGCTCTTCAACCTCTCC	5′-CTGTCTGGAGAGTGTAAGCAGG	56°C	35
**AC8**	5′-TCGGCTCTGGTCCTCATCAC	5′-GTTCTTCAAGGGTATCGACTTG	56°C	35
**AC9**	5′-GAGTTCGCCAAGGAGATGATGC	5′-TACAGGTAGGTCTTCATCTGGC	59°C	35
**µ-OR**	5′-ATGAAGACTGCCACCAACATCTAC	5′-GAAGAGAGGATCCAGTTGCAGAC	60°C	30
**δ-OR**	5′-GATGCGCTGGCCACCAGCAC	5′-GAACACGCAGATCTTGGTCAC	58°C	30
**κ-OR**	5′-ACATTGCCGTGTGCCACCCC	5′-TGCCACCACCACCAGGACCA	60°C	30
**GAPDH**	5′-CAAGGTCATCCATGACAACTTTG	5′-GTCCACCACCCTGTTGCTGTAG	58°C	30

### Radioligand Binding

Equilibrium binding studies were performed in triplicate determination for 2 h at RT in a total volume of 0.2 ml using the high-affinity µ-opioid receptor agonist [^3^H]DAMGO as the radioligand (2 nM) and 200–300 µg of membrane protein in TM buffer as described [19]. Non-specific binding was determined in the presence of 1 µM DAMGO. Reactions were stopped by rapid filtration over Whatman® GF/B glass fibre filters and radioactivity incorporated was determined by liquid scintillation in a Beckman LS6500 counter (Beckman Coulter). Maximum binding capacity was calculated according to the method of DeBlasi [20] using a *K_d_* of 1.4 nM for the human µ-opioid receptor [21].

### cAMP Accumulation

Cells grown in 24 well dishes were washed 3 times with DMEM containing 25 mM HEPES (DMEH) and 0.1 mM IBMX and then incubated for 30 min at 37°C. Cells were chilled on ice and media were replaced by DMEH containing the following stimulators and inhibitors of adenylyl cyclase: Forskolin (1 µM), Morphine (10 µM) and Naloxone (100 µM). Reactions were conducted for 15 min at 37°C and stopped by the addition of 0.75 ml 50 mM HCL per well. The amount of cAMP produced was measured by ELISA after acetylation of the samples.

### Cell Growth

Cells were cultured in 24 well plates for 5 (BT474) or 3 (SKBR3) days in insulin-free growth medium in the absence or presence of Morphine (10 µM), Naloxone (100 µM) and Heregulin (40 ng/ml) as described [22]. Cells were then washed twice with 0.5 ml PBS and fixed for 30 min at RT with 0.25 ml 20% methanol in PBS containing 0.1% crystal violet. The dye solution was aspirated, cells were washed extensively with PBS and finally solubilised for 2 h in 0.25 ml 0.1 M citric acid per well. Colour intensity was determined after 10 fold dilution at λ = 492 nm in a Tecan® Spectra Multiplate Reader. Background staining obtained from cells on the same plates fixed on day 0 was subtracted.

### Scratch Assay

Cell migration was evaluated using the in vitro scratch assay as described [23]. BT474 cells (4×10^5^/well) were seeded onto 6 well culture dishes containing poly-l-lysine-coated cover slips to yield 90% sub confluent monolayers after 5 d of culture in the absence (control) or presence of Heregulin (40 ng/ml), Morphine (10 µM) and Naloxone (100 µM) as indicated. Then, a scratch was made through the center of the well using a 200 µl pipette tip. Detached cells were removed and cells were cultured for another 24 h in complete medium containing the above ligands. Cells were then washed and fixed with 2% paraformaldehyde, before cell migration into the open area was assessed using an Olympus BH2 microscope.

### Cell Proliferation

DNA synthesis was determined for 2 h in 96 well plates using the BrdU Cell Proliferation ELISA (Roche Diagnostics) according to the manufactureŕs instruction. Media were removed and cells were stimulated for 2 h at 37°C with Heregulin β1 (40 ng/ml) and Morphine (10 µM) dissolved in 0.1 ml of insulin-deficient growth medium containing 10 µM BrdU. Reactions were stopped; cells were fixed and incubated with 0.1 ml anti-BrdU antibody coupled to horseradish peroxidase. After the addition of substrate, colour reaction was measurement at λ = 405 nm. All experiments were done in triplicate determination.

### ERK1/2 and Akt Phosphorylation

Cells grown in 12 well plates were washed and equilibrated for 30 min at 37°C in DMEH containing 0.1% BSA before cells were stimulated for 5 min with Heregulin-β1 (40 ng/ml), EGF (100 ng/ml), Morphine (10 µM) and Naloxone (100 µM) as indicated. Reactions were stopped on ice and cells were solubilized in 0.5 ml Laemmli sample buffer. In some experiments, the following protein kinase inhibitors were added during the equilibration period at the concentrations given: ErbB1 inhibitor AG1478 (5 µM), ErbB2 inhibitor AG825 (50 µM), PI3K inhibitor Wortmannin (1 µM) and the matrix metalloproteinase inhibitor EGCG (10 µM). The activation state of ERK1/2 and Akt was determined by Western blot using phospho-specific antibodies as described below.

### Apoptosis Assays

Stress-induced apoptosis was evaluated in 12 well dishes by culturing the cells for 6 h in serum-reduced growth medium (0.5% FCS) in the absence or presence of Morphine (10 µM), Naloxone (100 µM) and Heregulin (40 ng/ml). Reactions were stopped by removal of the medium and solubilisation of the cells in 0.5 ml Laemmli sample buffer. Apoptosis was determined by Western blot using an antibody recognizing cleaved PARP.

### Western Blot Analysis

Samples were cleared by centrifugation (10,000×g; 5 min) and proteins were resolved by electrophoresis over 8% (ErbB receptors) or 10% (Akt, ERK1/2, G proteins, PARP) polyacrylamide gels as described [6]. Proteins were then blotted onto PVDF-membranes, blocked and incubated overnight at 4°C with primary antibodies for phospho-specific ERK1/2 and Akt (1/2,000), ErbB receptors (1/1,000), PARP (1/1,000), G proteins (1/2,000) and β-tubulin (1/4,000) diluted in TBS/T/BSA (50 mM Tris-HCL, pH 8.0, 150 mM NaCl, 0.05% Tween 20, 0.1% BSA). Blots were washed, incubated with secondary peroxidase-conjugated IgG and developed using enhanced chemiluminescence (ECL). Immunoreactive bands were quantitated by video densitometry using the Herolab E.A.S.Y. RH-3 system.

### Determination of Protein Abundance and Immunoprecipitation

The presence of G proteins and ErbB receptors was determined in membranes using specific antibodies as described [6]. The formation of distinct ErbB receptor dimers was examined by immunoprecipitation experiments according to Garrett et al. [24] in cells chronically treated with Morphine (10 µM; 5 d) or not (control). Cells of a 75 cm^2^ flask were washed 3 times in PBS and stimulated for 10 min at 37°C with Morphine (10 µM) and Heregulin (40 ng/ml) in serum-free RPMI 1640 medium containing 0.1% BSA. Cells were then placed on ice, washed 3 times with PBS and cross-linked for 2 h with 5 mM DSP in PBS. Reactions were quenched with 10 mM Tris-HCl and membranes were prepared. Protein complexes were solubilized for 1 h at 4°C in TN buffer (10 mM Tris-HCl, 100 mM NaCl; pH 7.4) containing 1% Triton X 100, 1 mM phenylmethylsulfonyl fluoride and 4% Complete protease inhibitor cocktail (Roche Diagnostics). Samples were diluted 1/10 in TN buffer, cleared by centrifugation (10,000×g; 5 min) and equal amounts of solubilisates were subjected to immunoprecipitation overnight with anti-ErbB1 antibody (dilution 1/500) and 40 µl of Protein A-agarose beads. Immuncomplexes were washed 5 times with TN buffer containing 0.1% Triton X 100, cleaved by incubation for 5 min at 95°C in 0.1 ml of Laemmli sample buffer and subjected to gel electrophoresis.

### Cytochemistry

Induction of apoptosis was determined by Annexin V-FITC/propidium iodide staining of cells grown on poly-l-lysine-coated cover slips using the Annexin V-FITC Apoptosis Detection Kit (Bender MedSystems) as described [5]. Cells were then fixed for 30 min at RT with 2% paraformaldehyde in PBS before specimens were mounted using VectaMount ™ AQ Aqueous Mounting Medium (Vector Laboratories) and cells were examined by confocal microscopy (Carl Zeiss) using an 63×/1.4 oil immersion objective.

### Statistics

All data are expressed as the mean ± S.E. of the number or experiments given in the text. Statistical differences between two groups were analyzed by Student`s *t* test for unpaired data. Multiple comparisons were done by ANOVA followed by the Tukey post-hoc test.

## Results

### Characterization of Opioid Receptors in BT474 Cells

**Figure 1:**
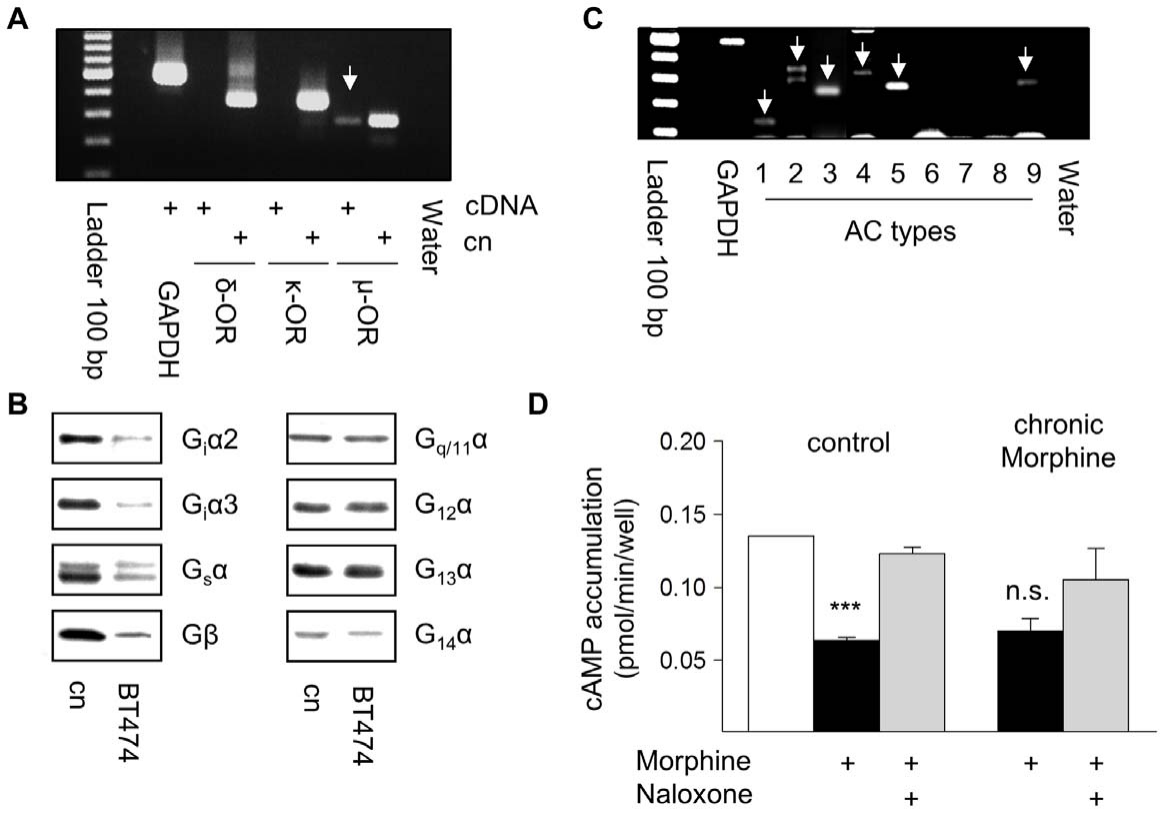
Characterization of µ-opioid receptors in BT474 cells. (**A**) Detection of µ-opioid receptors by RT-PCR. Reactions contained 10 ng of cDNA from BT474 cells or from the corresponding cloned receptors (Cn). Only a 293 bp fragment of the µ-opioid receptor is present (arrow). Control PCRs with primers for GAPDH or water instead of cDNA demonstrated the integrity of cDNA and the specificity of the RT-PCR reaction. Ladder: 100 bp. (**B**) Determination of G proteins in membranes from BT474 cells by Western blot using subtype-specific antibodies. BT474 cells contain inhibitory G_i_α2 and G_i_α3 as well as stimulatory G_s_α subunits. They also contain G_q/11_α, G_12_α, G_13_α, G_14_α and Gβ subunits. Membranes of MCF-7 cells served as control. (**C**) Identification of adenylyl cyclase (AC) isoforms in BT474 cells by RT-PCR. Reactions contained 10 ng of cDNA and primer pairs for all mammalian AC types. The following fragments were amplified (arrows): AC type 1 (143 bp), 2 (323 bp), 3 (259 bp), 4 (368 bp), 5 (312 bp), and 9 (325 bp). The quality of cDNA was verified by amplification of GAPDH, the specificity by using water instead of cDNA. Ladder: 100 bp. (**D**) Regulation of intracellular cAMP production by opioids. Control and chronically Morphine treated BT474 cells were stimulated for 15 min with Forskolin (10 µM). µ-Opioid receptor-mediated inhibition of cAMP production was assessed in the presence of Morphine (10 µM). Receptor specificity was determined by co-incubation with Naloxone (100 µM). The data shown are from 3 experiments. ***; significantly different at p<.001. n.s.; not quite significant.

To evaluate the specificity of acute and chronic opioid effects on human BT474 breast cancer cells, the presence of opioid receptors was determined first. RT-PCR experiments revealed the exclusive expression of µ-opioid receptors in this cell line (Fig. 1a). Receptor density was determined by equilibrium binding studies using the µ-opioid receptor selective peptide agonist [^3^H]DAMGO as the radioligand. The results show that BT474 cells contain about 7.3±2 fmol of µ-opioid receptors per mg of membrane protein (n = 5) and that chronic Morphine treatment does not affect receptor abundance (7.4±2 fmol per mg of membrane protein; n = 3). The ability of µ-opioid receptors to mediate cellular effects depends on the presence of adequate G proteins and effector systems. As shown in Fig. 1b, BT474 cell membranes carry diverse members of the family of inhibitory (G_i_α2, G_i_α3), stimulatory (G_s_α), Gq (G_q/11_α) and G12 (G_12_α, G_13_α, G_14_α) G proteins, allowing µ-opioid receptors to regulate multiple intracellular signalling pathways. Because they also express several adenylyl cyclase isoforms (AC 1–5, 9; Fig. 1c), the functional activity of µ-opioid receptors was assessed by their ability to regulate intracellular cAMP production. Indeed, acute µ-opioid receptor activation by Morphine (10 µM) significantly inhibits intracellular cAMP generation by about 53%, an effect almost completely blocked by Naloxone (100 µM). In chronically Morphine (10 µM, 5d)-treated cells an acute Morphine challenge is still able to inhibit cAMP production by about 48% (Fig. 1d). These results demonstrate that BT474 cells carry fully functional endogenous µ-opioid receptors and that chronic Morphine treatment does not desensitize µ-opioid receptor function providing an essential prerequisite to induce regulatory changes on post-receptor levels.

### Inhibition of Cell Growth and Migration by Morphine

**Figure 2:**
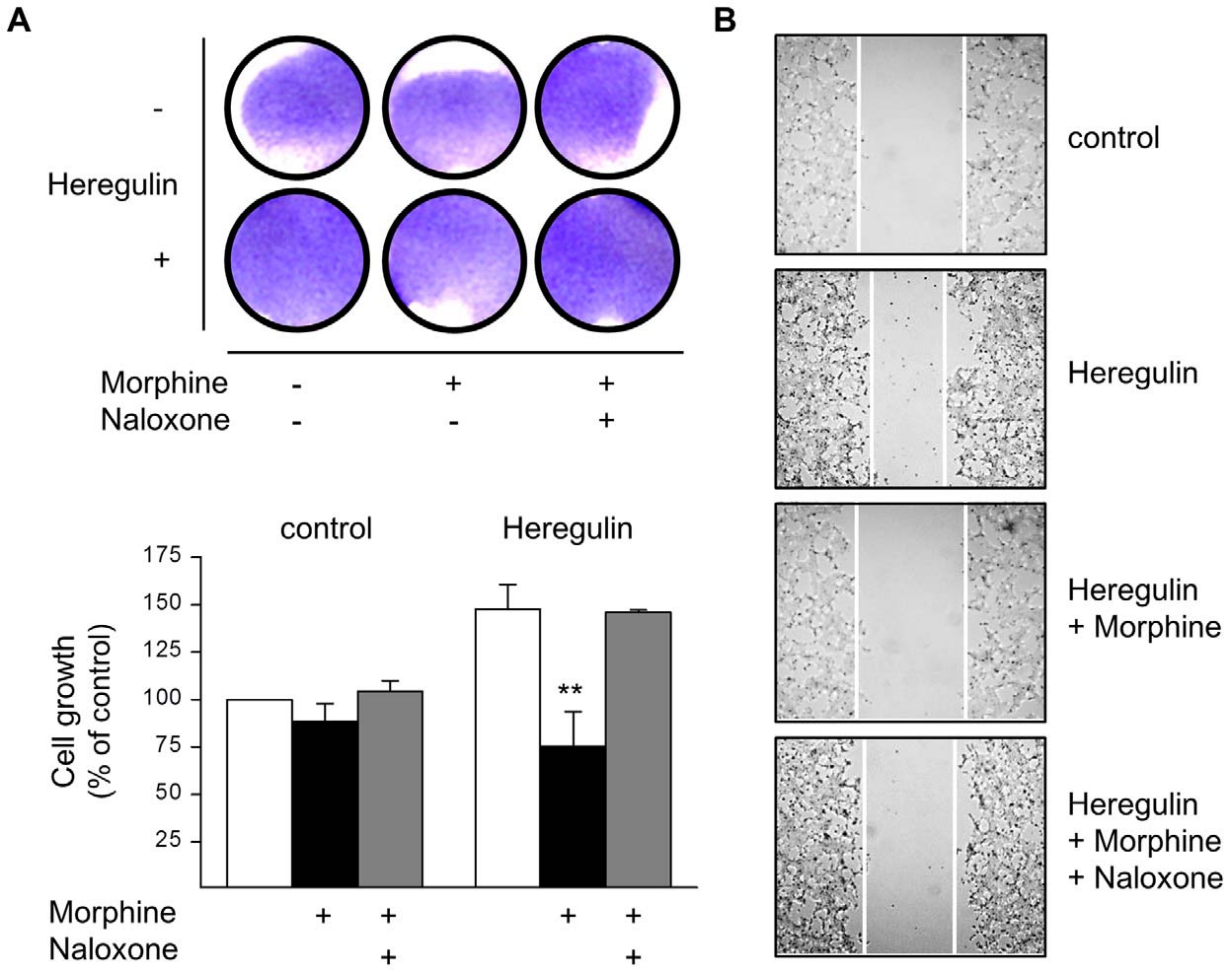
Regulation of BT474 cell growth and migration by Morphine. (**A**) BT474 cells were cultured for 5 d in the presence or absence of Morphine (10 µM), Naloxone (100 µM), and Heregulin (40 ng/ml), before cell growth was determined by crystal violet staining. Top: Photograph of tissue culture wells from a representative experiment before solubilisation of the dye. Bottom: Data of n = 6 independent experiments normalized to controls. Note that co-incubation of the cells with Morphine significantly attenuates Heregulin-stimulated cell growth (**, p<.005). (**B**) BT474 cell migration was assessed by the scratch assay done in cells grown for 5 d in the absence (control) or presence of Heregulin (40 ng/ml), Morphine (10 µM) and Naloxone (100 µM) as indicated. After scratching, cells were kept for another 24 h in the presence of the above ligands, before images were acquired using an Olympus BH-2 microscope (40× magnification). The figures shown are representative for 3 independent experiments yielding qualitatively similar results.

The growth of human BT474 breast cancer cells is driven by overexpressed ErbB2 receptor [11]. Due to the potential capacity of µ-opioid receptors to cross-regulate RTK signalling pathways [25], the ability of Morphine to interfere with BT474 cell growth was determined. Chronic exposure of the cells to Morphine slightly attenuates basal cell growth by about 11% as determined by crystal violet staining. However, the inhibitory opioid effect is much more pronounced when cells were cultured in the presence of the ErbB3 receptor agonist Heregulin. Chronic Heregulin treatment produces mitogenic effects and increases cell number by about 47%. Co-incubation of the cells with Morphine now significantly attenuates ErbB3-driven cell growth by about 49%. Inhibition of both basal and Heregulin-stimulated cell growth by Morphine is completely blocked by the opioid receptor antagonist Naloxone (Fig. 2a). In addition, treatment of BT474 cells with the inactive stereoselective enantiomer (+)-Morphine failed to affect cell growth (not shown), excluding a receptor-independent effect on mitochondrial pH due to the alkaloid structure of Morphine.

Opioids have been shown to regulate a number of different aspects of cell growth in tumour cell lines [26]. To gain a first indication as to whether Morphine might also interfere with Heregulin-stimulated BT474 cell migration, cell scratch assays were performed on cells growing on poly-l-lysine coated cover slips. As shown in Fig. 2b, Heregulin (40 ng/ml) treatment for 5 d produces morphological changes and induces cell migration into the gap 24 h after scratching. Co-incubation of the cells with Morphine (10 µM, 5d) prevents cell differentiation and migration into the open field, an effect that was partially blocked in the presence of Naloxone (100 µM). These results indicate that chronic Morphine treatment interferes with cell migration by preventing cell-matrix or cell-cell interactions.

### Regulation of Cell Proliferation by Morphine

**Figure 3:**
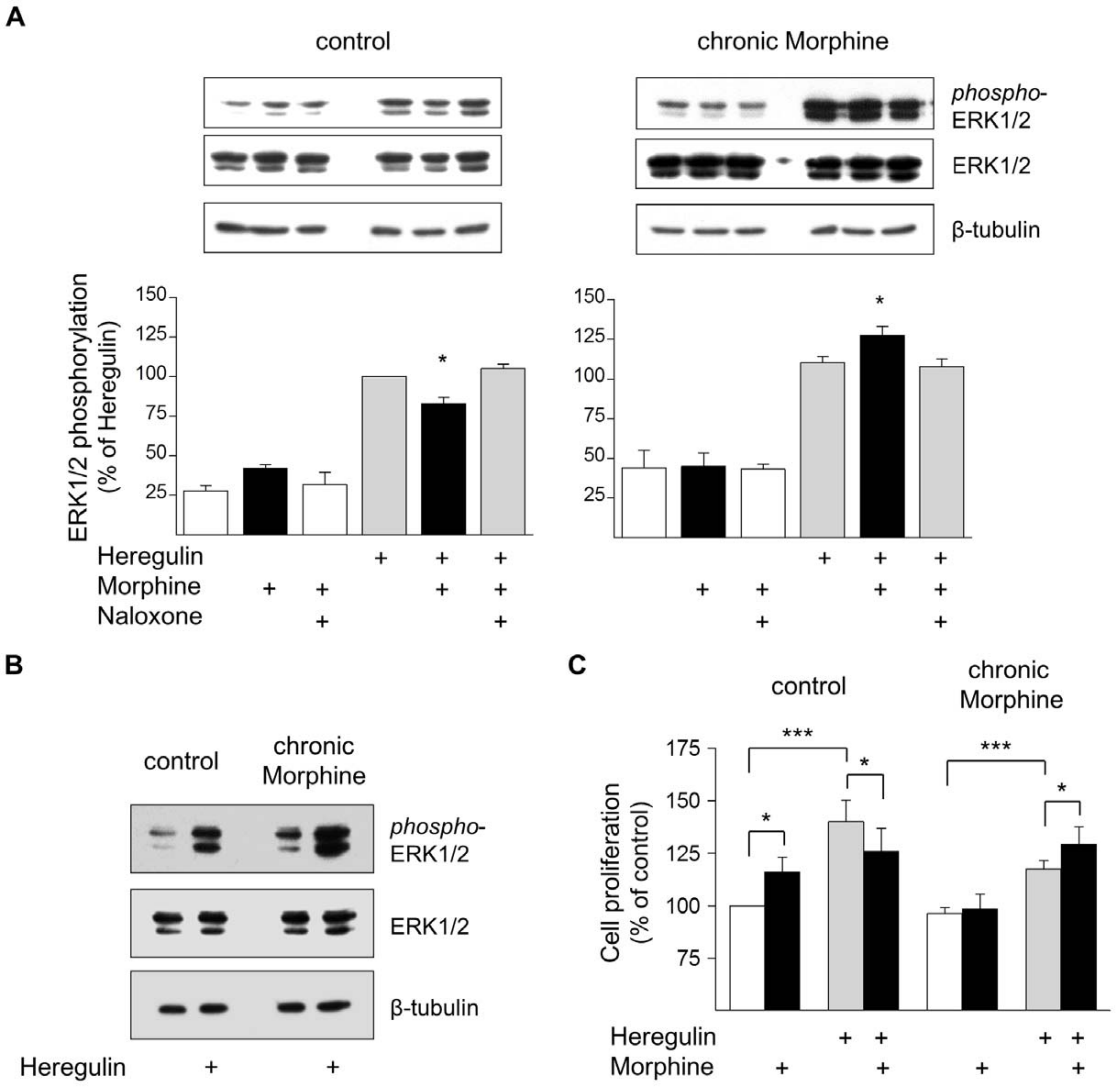
Regulation of BT474 cell proliferation by Morphine. (**A**) Determination of ERK1/2 activation in control and chronically Morphine (10 µM; 5d)-treated cells. Reactions were for 5 min at 37°C in the presence or absence of Morphine (10 µM), Naloxone (100 µM) and Heregulin (40 ng/ml), before ERK1/2 phosphorylation was determined by Western blot using a phospho-specific antibody. Samples from control and chronically Morphine-treated cells were run on the same gel. Immunoreactivity was quantified by video densitometry and normalized to Heregulin-stimulated values in control cells, which were set to 100%. Overall ERK1/2 abundance (42 and 44 kDa forms) was determined on the same samples using a pan reactive ERK1/2 antibody. Equal protein loading was verifies by staining with a β-tubulin antibody. Insets show representative Western blots. *; significantly different at p<.05 of n = 8 (control) and 6 (chronic Morphine) independent experiments. (**B**) Direct comparison of basal and Heregulin (40 ng/ml)-stimulated ERK1/2 activities in control and Morphine (10 µM; 5d)-treated cells. The same blot was stained for phospho-ERK1/2, pan-ERK1/2 and β-tubulin. Note that chronic morphine treatment elevates both basal and Heregulin (40 ng/ml)-stimulated ERK1/2 phosphorylation leaving overall ERK1/2 abundance unaffected. (**C**) Evaluation of cell proliferation by BrdU incorporation. Control and chronically Morphine (10 µM; 5 d)-treated cells were cultured for 2 h in the presence of 10 µM BrdU, before the amount incorporated was determined by ELISA. Data are from two independent experiments done in triplicate and normalized to controls, which were set 100%. *; statistically different at p<.05, ***; statistically different at p<.001.

Inhibition of BT474 cell growth by chronic Morphine treatment may be due to attenuation of cell proliferation and/or inhibition of cell survival. To discriminate between these two possibilities, the effect of acute and chronic Morphine treatment on ERK1/2 stimulation was determined. Most interestingly, acute µ-opioid receptor activation produces discrepant effects on ERK1/2 phosphorylation, depending on whether basal or Heregulin-stimulated activities were measured in control or chronically Morphine exposed cells. In control cells, acute µ-opioid receptor activation using a maximum effective concentration of Morphine (10 µM; determined in preliminary experiments) results in elevation of basal ERK1/2 phosphorylation, but significantly attenuates Heregulin (ErbB3)-induced ERK1/2 activation by about 17%. Chronic Morphine treatment (10 µM, 5d) results in elevation of basal and Heregulin-stimulated ERK1/2 phosphorylation. Under these conditions, acute µ-opioid receptor activation now significantly enhances ErbB3-stimulated ERK1/2 activity by about 14% (Fig. 3a). The increase in basal and Heregulin-stimulated ERK1/2 activity by chronic Morphine treatment is also given in Fig. 3b.

Similar results were observed when cell proliferation was examined by determination of DNA synthesis for 2 h using the BrdU assay. Again, acute µ-opioid receptor activation in control cells significantly stimulates basal and inhibits Heregulin-stimulated cell proliferation by about 16% and 10%, respectively. Chronic Morphine treatment completely abolishes the ability of acute µ-opioid receptor activation to stimulate basal cell proliferation and attenuates the overall effect of Heregulin by about 16%. In these cells, acute µ-opioid receptor activation now significantly enhances ErbB3-stimulated cell proliferation by nearly 10% (Fig. 3c). These results demonstrate that Morphine is able to regulate mitogenic signalling in human BT474 breast cancer cells and that the kind of acute µ-opioid receptor effect produced largely depends on the activation state of the cell. In addition, they also indicate that chronic Morphine treatment alters the ErbB receptor system controlling the growth of BT474 cells.

### Morphine Regulation of Akt Signalling

**Figure 4:**
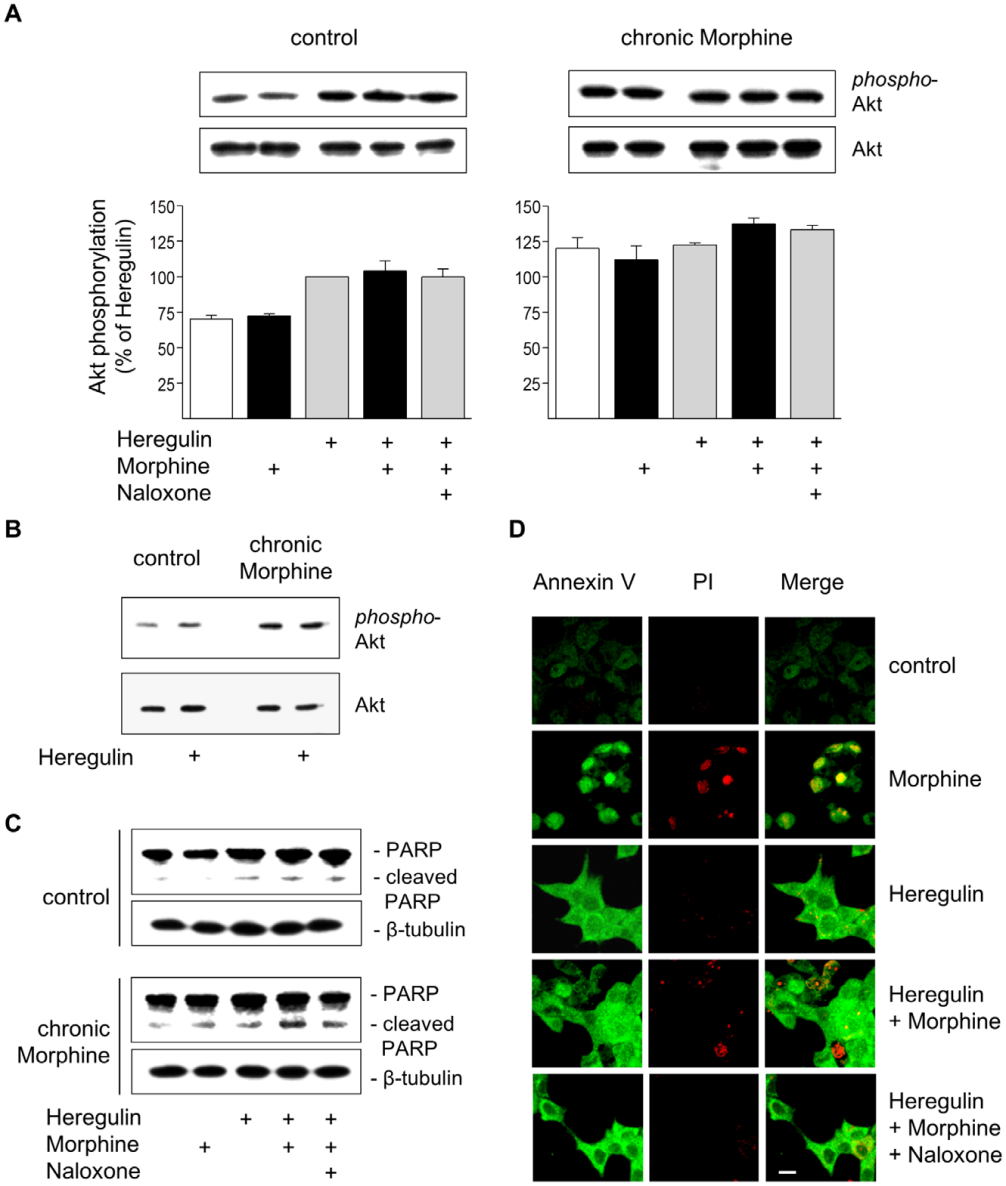
Regulation of cell survival and apoptosis by Morphine. (**A**) Determination of Akt activation in control and chronically Morphine (10 µM; 5d)-treated cells. Cells were incubated for 5 min at 37°C in the presence or absence of Morphine (10 µM), Naloxone (100 µM) and Heregulin (40 ng/ml), before Akt phosphorylation was determined by Western blot using a phosphor-specific antibody. The overall amount of Akt was determined by a phosphorylation-insensitive antibody. Insets show representative Western blots of the corresponding proteins (60 kDa) and β-tubulin (loading control). Immunoreactive bands were quantified and normalized to Heregulin-stimulated values in control cells, which was set to 100%. The data shown are from n = 4 independent experiments. (**B**) Comparison of basal and Heregulin (40 ng/ml)-stimulated Akt activation in control and Morphine (10 µM; 5d)-treated cells. Samples were run on the same gel and stained for phospho-Akt, total Akt and β-tubulin as loading control. Note that chronic morphine treatment increases basal and Heregulin (40 ng/ml)-stimulated levels of Akt phosphorylation. (**C**) Determination of PARP cleavage in BT474 cells. Cells were cultured in the absence (control) or presence of Morphine (10 µM; 5 d), before cells were washed and grown for an additional 6 h in serum-free Medium either in the absence or presence of Heregulin (40 ng/ml) Morphine (10 µM) and Naloxone (100 µM) as indicated. Samples were analysed by Western blot using an antibody recognizing full length (116 kDa) and cleaved (89 kDa) PARP. The same samples were blotted for β-tubulin (loading control). (**D**) Determination of apoptosis by Annexin V/propidium iodide staining. BT474 cells were cultured on coverslips for 5 d in the presence or absence of Morphine (10 µM), Naloxone (100 µM) and Heregulin (40 ng/ml) alone or in combination as indicated. Cells were sequentially stained with Annexin-FITC (green), propidium iodide (red), fixed and analysed by confocal microscopy. Bar: 20 µm.

The finding that chronic Morphine treatment attenuates basal and Heregulin-stimulated cell growth in the presence of an enhanced mitogenic µ-opioid receptor activity suggests that additional mechanisms must exist that shortens the lifespan of the cells. Therefore, we investigated whether chronic Morphine treatment would possibly attenuate cell survival by inhibition of Akt phosphorylation. As shown in !Fig. 4a, BT474 cells display basal levels of Akt phosphorylation, which are stimulated after activation of ErbB3 receptors by Heregulin. Chronic morphine treatment results in elevation of both basal and Heregulin-stimulated Akt activities. Acute µ-opioid receptor activation fails to affect basal and Heregulin-stimulated Akt signalling in both control and chronically Morphine-treated cells. The increase in basal and Heregulin-stimulated Akt activity after chronic Morphine treatment is demonstrated separately in Fig. 4b. These results demonstrate that chronic Morphine treatment increases basal and Heregulin-stimulated cell survival despite the overall attenuation of cell growth.

### Induction of Apoptosis by Chronic Morphine

To explain this apparent discrepancy, we examined the induction of apoptosis by chronic Morphine treatment by two different experimental approaches. We first compared the effect of Morphine on stress-induced apoptosis in control and chronically morphine treated BT474 cells by means of PARP cleavage. In control cells, serum starvation for 6 h failed to induce PARP cleavage. Apoptosis was induced only in the presence of Heregulin during the starvation period. Co-incubation of the cells together with Morphine had no further effect on PARP cleavage. Completely different results were obtained in chronically Morphine-treated cells. Here, serum starvation alone was already sufficient to induce PARP cleavage. The induction of apoptosis was further enhanced by the addition of Heregulin and Morphine, with their individual effects being additive (Fig. 4c). Similar results were obtained when apoptosis was determined by Annexin V/propidium iodide staining of the cells. Cultivation of BT474 cells on cover slips for 5 d in complete growth medium resulted in adherent patches that are negative for Annexin V-FITC staining. Addition of Morphine (10 µM) during the incubation period resulted in cell rounding, strong Annexin V binding and nuclear propidium iodide incorporation, which is indicative for the induction of apoptosis. As observed in the cell migration assay, chronic Heregulin treatment (40 ng/ml) produced morphological changes towards a more differentiated cell shape and induced of early steps of apoptosis as demonstrated by acquiring a polygonal cell shape and bright Annexin V-FITC staining. Concomitant treatment with Morphine prevented cell differentiation and induced apoptosis as indicated by cell rounding and nuclear propidium iodide incorporation. This chronic Morphine effect again is completely blocked by Naloxone (Fig. 4d). Together, these results demonstrate that chronic Morphine treatment of BT474 cells induces apoptosis and enhances Heregulin-stimulated cell fate.

### Chronic Morphine Treatment Alters ErbB Receptor Signalling

**Figure 5:**
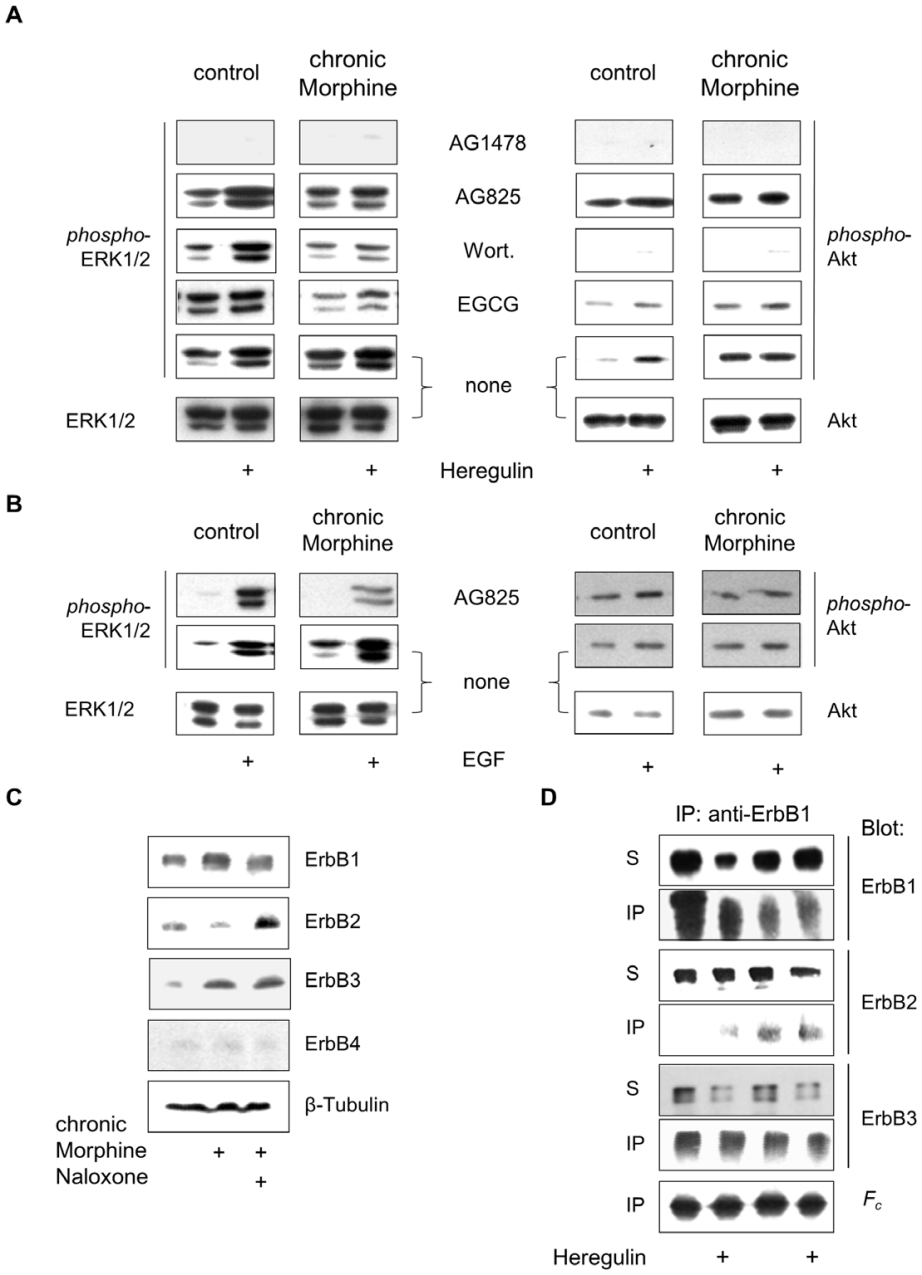
Analysis of chronic Morphine-induced changes in ErbB signalling pathways. (**A**) Effect of protein kinase blockers on basal and Heregulin-stimulated ERK1/2 and Akt phosphorylation. BT474 cells were cultured for 5 d in the absence (control) or presence of Morphine (10 µM), before the impact of ErbB1 (AG1478), ErbB2 (AG825), PI3K (Wortmannin; Wort.) and metalloproteinases (EGCG) on basal and Heregulin (40 ng/ml)-stimulated ERK1/2 and Akt signalling was determined by Western blot using phospho-specific antibodies. Controls were kept in the absence of protein inhibitors. To verify equal protein loading, controls were also stained with overall reactive anti-ERK1/2 and anti-Akt antibodies. (**B**) Involvement of ErbB2 on basal and EGF-stimulated ERK1/2 and Akt signalling. Controls and cells chronically exposed to Morphine (10 µM; 5d) were incubated with or without AG825 (50 µM; 30 min), before ERK1/2 and Akt phosphorylation was determined for 5 min in the absence or presence of EGF (100 ng/ml). Equal protein loading was verified by staining controls with overall reactive anti-ERK1/2 and anti-Akt antibodies. (**C**) Regulation of ErbB receptor abundance by chronic Morphine treatment in BT474 cells. Cells were cultured for 5 d in the presence or absence of Morphine (10 µM) and Naloxone (100 µM) as indicated and overall ErbB receptor levels were analysed by Western blot using specific antibodies for ErbB1 (175 kDa), ErbB2 (185 kDa), ErbB3 (185 kDa) and ErbB4 (170 kDa). Equal protein loading was verified by incubation of the blots with an antibody against β-tubulin. (**D**) Alteration of ErbB1 containing receptor dimers by chronic Morphine. Controls and chronically Morphine (10 µM; 5 d)-treated BT474 cells were stimulated for 5 min with or without Heregulin (40 ng/ml) to form receptor dimers. Proteins were cross-linked and ErbB1 containing dimers were immunoprecipitated using an anti-ErbB1 antibody. Individual ErbB receptors were determined in whole cell solubilisates (S) and immunoprecipitates (IP) by Western blot using type-specific antibodies. Equal protein load was verified by determination of IgG heavy chains (*F_c_*).

The growth characteristics of cancer cells are determined by the signalling strength and duration of individual ErbB receptor homo- and heterodimers [9]. To obtain a first indication for whether changes in the ErbB receptor network would account for the differences observed in mitogenic µ-opioid receptor signalling after chronic Morphine treatment, several components of receptor tyrosine kinase-stimulated ERK1/2 and Akt signalling pathways were analysed by the use of established protein kinase inhibitors. ErbB2-overexpressing BT474 cells contain physiologic levels of ErbB3 receptors that are essential for ErbB2-driven cell growth [27]. To our surprise, inhibition of catalytic ErbB1 function by the tyrphostin analogue AG 1478 completely blocked all aspects of basal, Morphine- and Heregulin-stimulated ERK1/2 and Akt phosphorylation in both control and chronically morphine treated cells, indicating that ErbB1 must represent the obligatory binding partner in all ErbB receptor dimers. In control cells, blockade of ErbB2 receptor function by AG825 had no effect on basal and Heregulin-stimulated ERK1/2 and Akt signalling. In contrast, AG825 exclusively attenuated Heregulin-stimulated ERK1/2 activation in chronically Morphine-treated cells. Because AG825 also fails to affect Heregulin-stimulated Akt signalling, the ErbB1/ErbB2 heterodimer appears to mediate ErbB3-stimulated ERK1/2 signalling in Morphine-treated cells. Pre-incubation of the cells with the PI3K inhibitor Wortmannin completely blocks Akt phosphorylation under all conditions. In control cells, Wortmannin has no effect on basal and Heregulin-stimulated ERK1/2 phosphorylation. However, it largely attenuates ErbB3-stimulated ERK1/2 activation in chronic Morphine-treated cells. This result implies that chronic Morphine treatment of BT474 cells switches Heregulin-stimulated ERK1/2 signalling from a PI3K-independent to a PI3K-dependent mechanism. The contribution of metalloproteinases on chronic Morphine-induced mitogenic signalling was finally evaluated by pre-incubation of the cells with EGCG. To our surprise, blockade of ectodomain shedding by EGCG selectively attenuates basal and Heregulin-induced ERK1/2 phosphorylation in chronically Morphine-treated, but not in control cells. In addition, overall levels of Akt phosphorylation appear to be reduced as compared to controls. This finding indicates that activation of ErbB1/ErbB2 heterodimers in chronically Morphine-treated cells appears to require the release of an autocrine factor (Fig. 5a). Further studies revealed that the IGF-1 receptor fails to contribute to chronic Morphine-induced changes in ERK1/2 and Akt signalling (not shown).

To verify whether ErbB1/ErbB2 heterodimers contribute to mitogenic signalling in chronically Morphine-treated BT474 cells, these were pre-treated with AG825 and subsequently stimulated with EGF. In both control and chronically Morphine-treated cells, activation of ErbB1 by EGF leads to stimulation of ERK1/2 and Akt activities, as previously observed for Heregulin. Again, functional inactivation of ErbB2 by AG825 selectively attenuates EGF-stimulated ERK1/2 phosphorylation in Morphine-treated cells (Fig. 5b). Thus, EGF appears to induce the formation of ErbB1/ErbB2 heterodimers only after chronic Morphine treatment of the cells.

One major mechanism by which the formation of ErbB receptor dimers may be modulated is by regulation of receptor quantity. As shown in Fig. 5c, chronic Morphine treatment differentially regulates the relative abundance of ErbB receptor types present. By means of Western blot analysis of BT474 plasma membranes, strong immunoreactive bands were obtained for ErbB1, ErbB2 and ErbB3 receptors. The ErbB4 receptor signal was only barely detectable. Chronic Morphine treatment produces complex changes in ErbB receptor levels. It increases the relative abundance of ErbB1 and ErbB3 and strongly down-regulates the amount of ErbB2. The levels of ErbB4 remained unchanged. Concomitant treatment of the cells with Naloxone at least partly prevented these changes. These results suggest that chronic Morphine treatment could possibly alter the formation of individual ErbB homo- and heterodimers by regulating ErbB receptor abundance.

To test whether chronic Morphine indeed leads to the rearrangement of ErbB receptor dimers, co-precipitation studies using an anti-ErbB1 antibody were performed. In membranes from control cells, ErbB1 appears to exclusively form heterodimers with ErbB3. Short-term stimulation of the cells with Heregulin does not affect ErbB1/ErbB3 heterodimers, but leads to co-precipitation of traces of ErbB2. Besides ErbB1/ErbB3 heterodimers, chronic Morphine treatment also results in the formation of ErbB1/ErbB2 heterodimers. Again, short-term stimulation of the cells with Heregulin did not affect dimer formation (Fig. 5d). Together, these results demonstrate that chronic Morphine treatment alters ERK1/2 and Akt signalling in BT474 cells by the establishment of newly formed ErbB1/ErbB2 heterodimers.

**Figure 6:**
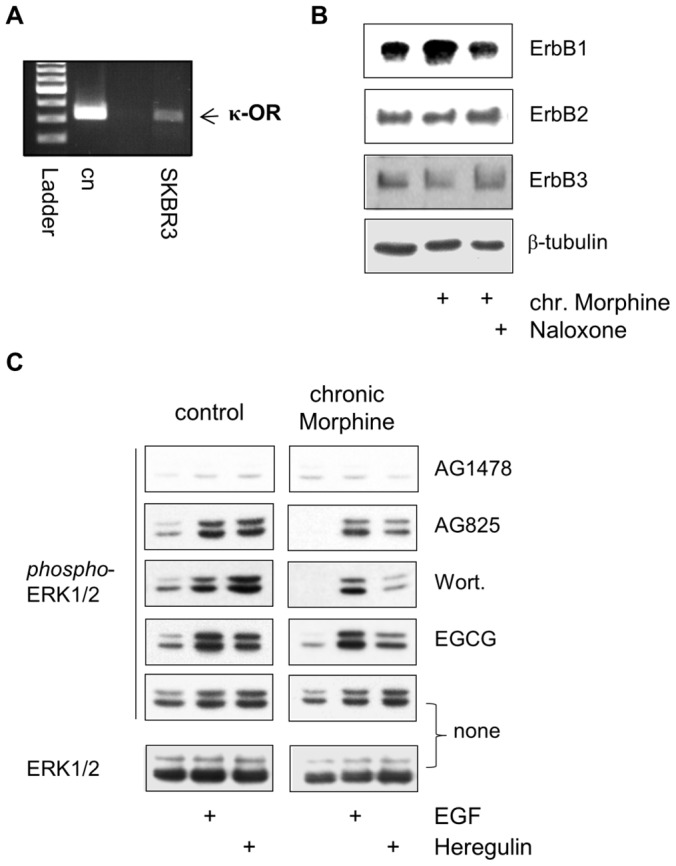
Regulation of ErbB signalling by chronic Morphine in SKBR3 cells. (**A**) Identification of κ-opioid receptors in SKBR3 human mammary adenocarcinoma cells by RT-PCR. Reactions contained 10 ng of cDNA from SKBR3 cells or plasmid pcDNA3.1 containing the cloned receptor. A 322 bp fragment of the κ-opioid receptor is present in the cells (arrow). Ladder: 100 bp. (**B**) Regulation of ErbB receptor abundance by chronic Morphine treatment. Cells were cultured for 3 d in the presence or absence of Morphine (10 µM) and Naloxone (100 µM), before membranes were prepared and ErbB receptors were determined by Western blot using antibodies specific for ErbB1 (175 kDa), ErbB2 (185 kDa) and ErbB3 (185 kDa). Equal protein loading was determined using an antibody against β-tubulin. (**C**) Effect of chronic Morphine treatment on ErbB1- and ErbB3-stimulated ERK1/2 signalling. SKBR3 cells were cultured for 3 d in the absence (control) or presence of Morphine (10 µM), before the impact of protein inhibitors AG1478 (ErbB1), AG825 (ErbB2), Wortmannin (Wort; PI3K) and EGCG (metalloproteinases) on basal, EGF (100 ng/ml)- and Heregulin (40 ng/ml)-stimulated ERK1/2 phosphorylation was determined by Western blot. Controls were kept in the absence of protein inhibitors (none). Total ERK1/2 was determined using an overall reactive antibody.

To investigate whether similar alterations are also induced in other ErbB-driven breast tumour cells, the effect on chronic Morphine treatment on mitogenic signalling was examined in SKBR3 human mammary carcinoma cells. These cells carry high levels of ErbB2 and physiologic levels of ErbB1 and ErbB3 [16]. RT-PCR experiments verified the expression of κ-type opioid receptors in this cells system (Fig. 6a), providing the basis for the development of chronic Morphine-induced adaptations at the level of the ErbB network. Although chronic Morphine (10 µM, 3d) treatment produced a subtle increase in the abundance of ErbB1 in plasma membranes (Fig. 6b), no major effect on cell growth was found as assessed by crystal violet staining (100 vs. 103.8±6%, mean ± SE, n = 5). Nonetheless, the use of protein kinase inhibitors revealed the induction of profound alterations in ErbB3-stimulated ERK1/2 signalling by chronic Morphine treatment. As shown in Fig. 6c, EGF (100 ng/ml) and Heregulin (40 ng/ml) both stimulate ERK1/2 phosphorylation to about the same extent in control and chronically Morphine (10 µM, 3d)-treated cells. In both cells, pre-treatment with the ErbB1 inhibitor AG1478 not only attenuates ErbB1-, but also ErbB3-stimulated ERK1/2 activation. This indicates that Heregulin-stimulated ERK1/2 signalling requires ErbB1 in both control and chronically Morphine-treated SKBR3 cells. In contrast, pre-incubation of the cells with AG825, Wortmannin and EGCG specifically attenuated ErbB3-stimulated ERK1/2 activation in chronically Morphine-treated, but not in control cells. Thus, chronic Morphine treatment of SKBR3 cells switches Heregulin-mediated ERK1/2 signalling to a PI3K-, matrix metalloproteinase- and ErbB2-dependent mechanism comparable to that observed in BT474 cells. These results demonstrate that in tumour cells the dysregulated ErbB receptor system represents a target for chronic opioid treatment.

## Discussion

There is increasing evidence that opioid analgesics interfere with several aspects of tumour growth [3]. Here we describe that human BT474 breast cancer cells endogenously express functionally active µ-opioid receptors and that chronic Morphine treatment produces fundamental changes in the ErbB receptor network resulting in attenuation of Heregulin-stimulated cell growth. These data add a new dimension on the mechanism of opioid-stimulated mitogenic signalling, as they demonstrate that opioid receptors not only transactivate RTK-associated signalling pathways but also may alter the growth characteristics of tumour cells by rearrangement of the ErbB receptor system.

The ErbB receptor family integrates a number of auto- and paracrine signals to regulate normal cell growth, differentiation and development [28]. In many cancers, the ErbB receptor network is hyperactive and leads to clonal expansion of tumour cells [29]. Among the best studied examples are human breast cancers that overexpress the ErbB2 receptor. Because the level of ErbB2 gene amplification in ErbB2 positive cancers is much higher than in normal tissue, targeted inactivation of the ErbB2 protein evolved as one to the most promising and successful antineoplastic strategies in these cancers. The outcome of anti-ErbB2 treatments, however, is often limited by the induction of compensatory resistance mechanisms due to the high plasticity of the ErbB network [17]. In this context, the present study performed on human BT474 and SKBR3 breast cancer cells opens a new perspective in order to enhance the sensitivity of ErbB2-directed treatments and to avoid escape mechanisms. This idea bases on the finding that chronic Morphine treatment induces apoptosis in BT474 cells by affecting the specificity of Heregulin-stimulated ErbB signalling. These effects are mediated by the formation of ErbB1/ErbB2 heterodimers in chronically Morphine treated cells that switch tumour cell growth from an ErbB3- to and ErbB2-dependent mechanism. Opposite changes in ErbB receptor plasticity have been previously reported for BT474 and SKBR3 mamma carcinoma cells chronically treated with specific anti-ErbB1 and ErbB2 inhibitors. In these studies, inactivation ErbB1 and ErbB2 receptors restored cell growth by reactivation of ErbB3 and ErbB4 receptor signalling [30] and redirected tumour cell growth from an ERK1/2 to an Akt-dependent mechanism [24].

The changes in ErbB receptor network by chronic Morphine treatment were detected by the use of protein kinase inhibitors to block acute µ-opioid receptor-stimulated ERK1/2 and Akt activity. Although the single use of protein kinase inhibitors may be endowed with several drawbacks, like non-specific effects, it was not possible to verify the present results by genetic approaches because siRNA-mediated knock-down or protein overexpression per se may alter ErbB signalling and mask possible chronic Morphine effects. Nevertheless, the use of validated and generally accepted protein kinase inhibitors in this study provided novel insight into the composition of ErbB receptor dimers in BT474 and SKBR3 cells. Our experiments suggest that ErbB1 appears to represent the obligatory binding partner in ErbB receptor dimers, because pre-treatment of the cells with AG1478 blocked all aspects of basal and Heregulin-stimulated ERK1/2 and Akt activities. To our surprise, ERK1/2 signalling was exclusively affected by AG825 in chronically Morphine treated, but not in control cells. This suggests that chronic Morphine treatment induces the formation of ErbB1/ErbB2 heterodimers, which was verified by our co-immunoprecipitation studies in BT474 cells. Co-immunoprecipitation also revealed the existence of ErbB1/ErbB3 heterodimers in both naïve and chronically Morphine-treated cells, substantiating the notion that the growth of BT474 cells is dependent on ErB3 signalling despite overexpression of ErbB2 [27].

**Figure 7:**
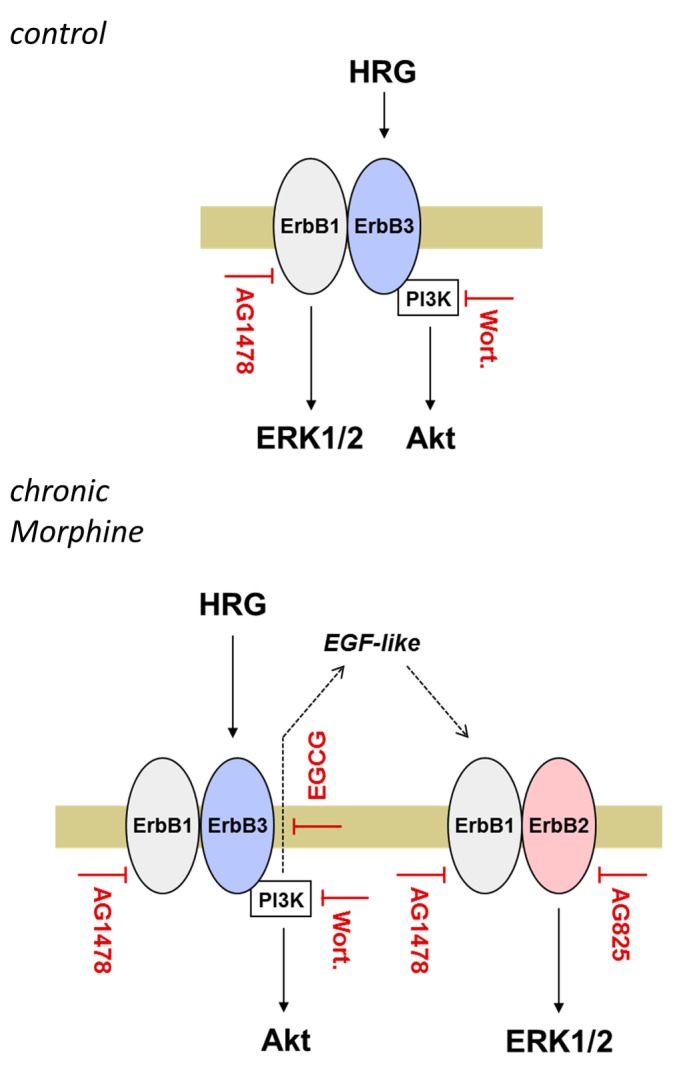
Model of chronic Morphine-induced changes in ErbB signalling. The scheme depicts the differences in Heregulin-stimulated ErbB signalling in control and chronically Morphine-treated BT474 cells. In control cells, stimulation with Heregulin leads to activation of ERK1/2 and Akt signalling via ErbB1/ErbB3 heterodimers. While AG1478 blocks both Heregulin-stimulated ERK1/2 and Akt signalling, inhibition of PI3K by Wortmannin specifically prevents Akt signalling. Because ErbB3 phosphorylation and PI3K activation is dependent on the presence of a dimerized ErbB member with catalytic activity, these results indicate that Heregulin-stimulated ERK1/2 signalling is predominantly mediated through ErbB1. The failure of Wortmannin to affect ERK1/2 activation in control cells further implicates that Heregulin stimulates Akt signalling via ErbB3. Chronic Morphine treatment alters mitogenic signalling by rearrangement of ErbB heterodimers. Whereas Heregulin still stimulates Akt signalling via ErbB1/ErbB3, ERK1/2 signalling is now accomplished by ErbB1/2 heterodimers. These are activated indirectly by an EGF-like ligand liberated from Heregulin in a PI3K (Wortmannin)- and metalloproteinase (EGCG)-dependent manner.

To investigate the role of chronic Morphine treatment on ErbB3 signalling, Heregulin stimulated ERK1/2 and Akt signalling was investigated in BT474 and SKBR3 cells. In control cells, Heregulin appears to mediate ERK1/2 and Akt signalling via activation of single ErbB1/ErbB3 heterodimers. Activation of ErbB3 in ErbB1/ErbB3 heterodimers has been shown to mediate signal transduction by transactivation of ErbB1, which in turn stimulates the Ras/Raf/ERK1/2 cascade by binding the adapter proteins Shc and Grb2 [31]. Because ErbB3 is deficient of kinase activity, Heregulin-stimulated Akt signalling must be accomplished by Grb2/Gab1 mediated cross-regulation of PI3K [32]. After the introduction of ErbB1/ErbB2 heterodimers, Heregulin-stimulated ERK1/2, but not Akt signalling became dependent on ErbB2 receptors in BT474 cells. This finding suggests that Akt signalling is further accomplished by ErbB1/ErbB3 heterodimers, whereas ERK1/2 signalling is redirected to ErbB1/ErbB2 heterodimers. Such a mechanism would require the autocrine release of an EGF-like ligand by Heregulin, which then transactivates the ErbB1/ErbB2 heterodimer. In our experiments, blockade of metalloproteinase activity by EGCG largely attenuated Heregulin- and EGF-stimulated ERK1/2 activation, indicating that Heregulin indeed induces the release of an EGF-like ligand. Reactivation of a comparable autocrine loop maintaining ErbB2 activity has been previously described for breast cancer cells chronically treated with an anti-ErbB2 directed antibody [!33]. In chronically Morphine treated cells, Heregulin-stimulated ERK1/2 signalling also became dependent on PI3K, whereas pre-treatment with Wortmannin is ineffective in control cells. To date, cross-activation of ERK1/2 signalling by PI3K has been observed only under distinct conditions, however the underlying cellular mechanism remain unclear. It has been suggested that PI3K-dependent Ras/Raf/ERK1/2 signalling is that ErbB1 recruits GAP/SH2 complexes to cell membranes by PIP3 in the presence of low doses of EGF-like ligands [34]. Although we cannot exclude such a mechanism, our results suggest that PI3K contributes to the Heregulin-induced cleavage of EGF-like ligands by metalloproteinase. This would be in line with the finding that PI3K is able to block a negative feedback loop in ErbB3-driven tumour cells, resulting in a compensatory activation of the ERK signalling pathway [35]. The question remains how Heregulin brings about Akt phosphorylation in chronically Morphine treated BT474 cells. Despite the lack of catalytic activity, ErbB3 is able to directly activate PI3K by binding all three regulatory subunits through their SH-domains [13]. However, phosphorylation of the 6 consensus phosphorylation sites in the C-terminus of ErbB3 is only induced after ligand-induced activation of an ErbB binding partner. In this respect, an EGF-like ligand released by Heregulin might activate accomplish direct Akt signalling via ErbB1/ErbB3 heterodimers in chronically Morphine-treated BT474 cells. Such a mechanism appears plausible because blockade of metalloproteinase activity in chronically Morphine-treated cells substantially attenuates the levels of Akt phosphorylation. A model of chronic Morphine-induced changes in Heregulin-stimulated ERK1/2 and Akt signalling is shown in !Fig. 7.

In BT474 cells, the functional consequence of the chronic Morphine-induced alterations in ErbB signalling is the enhancement of Heregulin-induced apoptosis. Morphine is known to possess pro-apoptotic effects in a number or cell models. Pathways proposed to be involved in Morphine-induced apoptosis include an increased expression of pro-apoptotic Bim, decreased expression of anti-apoptotic Bcl-2 [36] and stabilization of p53 [37]. On the other hand opioids have also been shown to display strong anti-apoptotic properties in a number of cell models, which are mediated by activation of the Akt pathway [5]. On the first view, the induction of apoptosis in BT474 cells is somewhat surprising, since chronic Morphine treatment consistently increased basal levels of Akt phosphorylation. One possible explanation for this apparent discrepancy is that the switch from an ErbB3- to an ErbB2-dependent signalling attenuates cell survival by masking ErbB3 driven Akt signalling. An opposite mechanism has been described in cancer cells with dominant PI3K signalling in which a negative feedback loop dampens compensatory Ras/Raf/ERK1/2 activity, maintaining anti-apoptotic signalling [35]. In this respect, chronic Morphine-induced ErbB2 signalling may be viewed as an escape mechanism that blocks Akt signalling.

The mechanism by which chronic Morphine regulates the rearrangement of ErbB receptor dimers remains to be determined. One possible explanation would be that Morphine, which acts as a partial agonist at the µ-opioid receptor, produces persistent inhibitory opioid signalling due to its failure to desensitize the receptor [38]. Indeed, although present at relatively low levels in BT474, chronic Morphine treatment had no effect on µ-opioid receptor abundance and signalling, providing an absolute requirement for the induction of adaptational changes on post-receptor level. In neuronal cells, such changes are proposed to contribute to the development of opioid tolerance and dependence [1]. In non-neuronal cells, chronic Morphine treatment has been shown to facilitate persistent ERK1/2 signalling by down-regulation of c-Cbl and blockade of ErbB1 receptor degradation [18]. Inhibition of protein degradation could explain our finding that chronic Morphine treatment elevates the abundance of ErbB1 and ErbB3 in BT474 cells. An opposite down-regulation of ErbB2 after chronic Morphine treatment might be the result of activation-induced cleavage of the receptor, which is typical for ErbB2-overexpressing cells [39]. Because regulation of ErbB abundance is a critical determinant for the signalling activity in a given cell system [28], quantitative changes in ErbB receptors represent a potential mechanism by which chronic Morphine treatment influences the composition of individual ErbB dimers.

### Conclusions

The present study demonstrates that chronic Morphine treatment produces fundamental changes in the ErbB receptor network of human breast cancer cells that are characterized by quantitative alterations in the abundance of individual ErbB receptors, alterations in ErbB dimer formation and the introduction of an autocrine loop that redirects Heregulin-stimulated ERK1/2 activation from ErbB1/ErbB3 to ErbB1/2-heterodimers. Although these changes produce no major effect on Morphine stimulated cell growth and mitogenic signalling, they become evident when Heregulin-stimulated effects on cell growth were examined. Direct modulation of ErbB signalling mechanisms could explain the many discrepant opioid effects on cell growth and apoptosis observed so far in individual cell systems and may provide a novel strategy to stabilize certain ErbB dimers in order to enhance the sensitivity of ErbB-directed anti-tumour therapies and to avoid the development of drug resistance by preventing escape mechanisms.
