# Correction: Chronic Morphine Treatment Attenuates Cell Growth of Human BT474 Breast Cancer Cells by Rearrangement of the ErbB Signalling Network

**DOI:** 10.1371/journal.pone.0140727

**Published:** 2015-10-09

**Authors:** Inka Regine Weingaertner, Sarah Koutnik, Hermann Ammer

There is an undisclosed splice in Figure 1C in the published paper. In order to provide a clearer version of Figure 1C, the authors cropped the original image of the gel and spliced in a reformatted version of the 3rd AC type lane. The uncropped, original image of the gel is provided here as S1 Image.

Additionally, there is an error in Fig 2B. The "control" panel and "Heregulin + Morphine" panel are mistakenly from the same sample. The authors have provided a correct version of Fig 2, which includes a new image for the "control" panel. The uncropped samples for Fig 2B are provided here as S2 Image.

**Fig 2:**
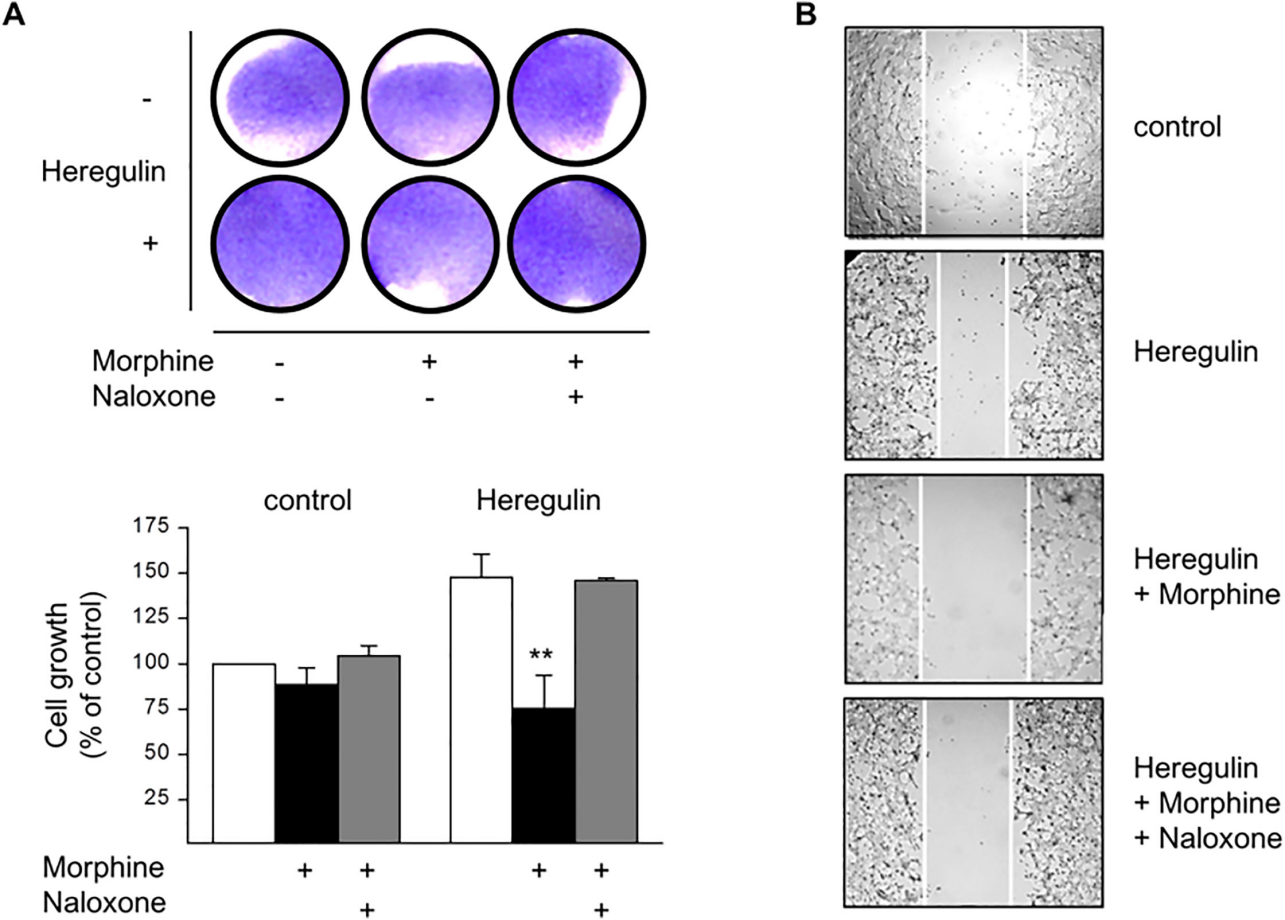
Regulation of BT474 cell growth and migration by Morphine. (**A**) BT474 cells were cultured for 5 d in the presence or absence of Morphine (10 μM), Naloxone (100 μM), and Heregulin (40 ng/ml), before cell growth was determined by crystal violet staining. Top: Photograph of tissue culture wells from a representative experiment before solubilisation of the dye. Bottom: Data of n = 6 independent experiments normalized to controls. Note that co-incubation of the cells with Morphine significantly attenuates Heregulin-stimulated cell growth (**, p < .005). (**B**) BT474 cell migration was assessed by the scratch assay done in cells grown for 5 d in the absence (control) or presence of Heregulin (40 ng/ml), Morphine (10 μM) and Naloxone (100 μM) as indicated. After scratching, cells were kept for another 24 h in the presence of the above ligands, before images were acquired using an Olympus BH-2 microscope (40× magnification). The figures shown are representative for 3 independent experiments yielding qualitatively similar results.

## Supporting Information

S1 Image Uncropped, original image of gel for Figure 1C. (TIF)

S2 Image Uncropped samples for Fig 2B. (TIF)
