# Correction: Chronic Morphine Treatment Attenuates Cell Growth of Human BT474 Breast Cancer Cells by Rearrangement of the ErbB Signalling Network

**DOI:** 10.1371/journal.pone.0153824

**Published:** 2016-04-14

**Authors:** Inka Regine Weingaertner, Sarah Koutnik, Hermann Ammer

There are undisclosed splices between lanes 2 and 3 for each panel in Fig 4A. Because no acute opioid effect on Akt phosphorylation was observed in naïve cells, the authors spliced out the Naloxone control band in order to save space. The authors have provided a corrected version of Fig 4, in which all splices are clearly demarcated with a vertical black line. The raw blots for each panel in Fig 4A are provided below as supporting information.

**Fig 4:**
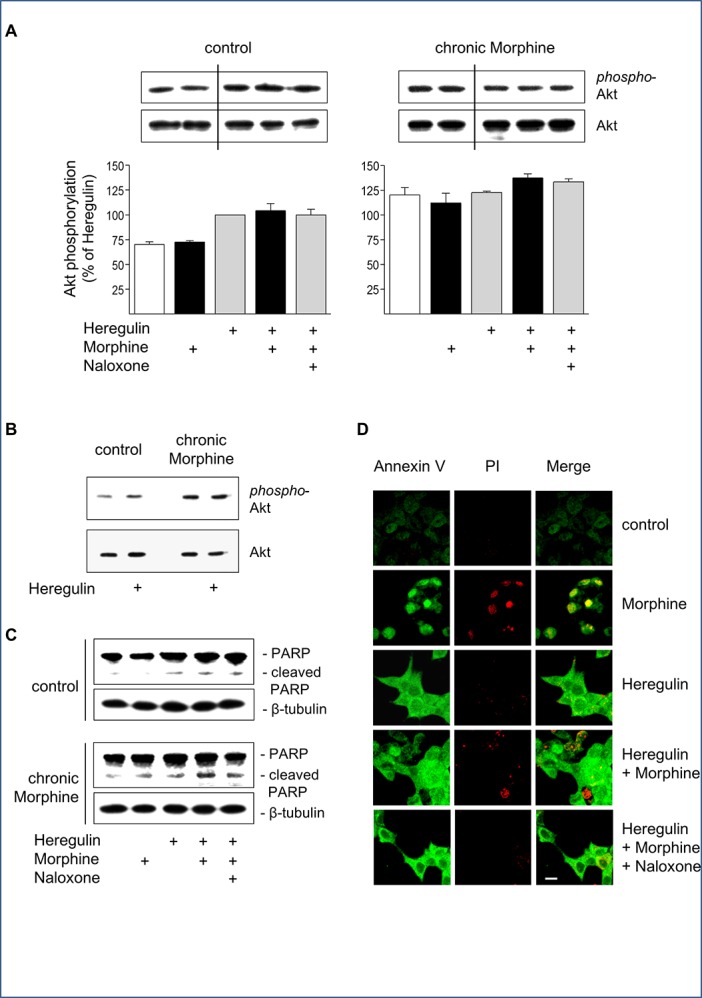
Regulation of cell survival and apoptosis by Morphine. (**A**) Determination of Akt activation in control and chronically Morphine (10 μM; 5d)-treated cells. Cells were incubated for 5 min at 37°C in the presence or absence of Morphine (10 μM), Naloxone (100 μM) and Heregulin (40 ng/ml), before Akt phosphorylation was determined by Western blot using a phosphor-specific antibody. The overall amount of Akt was determined by a phosphorylation-insensitive antibody. Insets show representative Western blots of the corresponding proteins (60 kDa) and β-tubulin (loading control). Immunoreactive bands were quantified and normalized to Heregulin-stimulated values in control cells, which was set to 100%. The data shown are from n = 4 independent experiments. (**B**) Comparison of basal and Heregulin (40 ng/ml)-stimulated Akt activation in control and Morphine (10 μM; 5d)-treated cells. Samples were run on the same gel and stained for phospho-Akt, total Akt and β-tubulin as loading control. Note that chronic morphine treatment increases basal and Heregulin (40 ng/ml)-stimulated levels of Akt phosphorylation. (**C**) Determination of PARP cleavage in BT474 cells. Cells were cultured in the absence (control) or presence of Morphine (10 μM; 5 d), before cells were washed and grown for an additional 6 h in serum-free Medium either in the absence or presence of Heregulin (40 ng/ml) Morphine (10 μM) and Naloxone (100 μM) as indicated. Samples were analysed by Western blot using an antibody recognizing full length (116 kDa) and cleaved (89 kDa) PARP. The same samples were blotted for β-tubulin (loading control). (**D**) Determination of apoptosis by Annexin V/propidium iodide staining. BT474 cells were cultured on coverslips for 5 d in the presence or absence of Morphine (10 μM), Naloxone (100 μM) and Heregulin (40 ng/ml) alone or in combination as indicated. Cells were sequentially stained with Annexin-FITC (green), propidium iodide (red), fixed and analysed by confocal microscopy. Bar: 20 μm.

Additionally, there is an error in Fig 5A that was introduced during figure preparation. In the right half of Fig 5A, the lowest control panel is erroneously a duplicate of the first two lanes of the lower control panel in Fig 4A. The authors have provided a corrected version of Fig 5. The raw blots for Fig 5A are provided below as supporting information.

**Fig 5:**
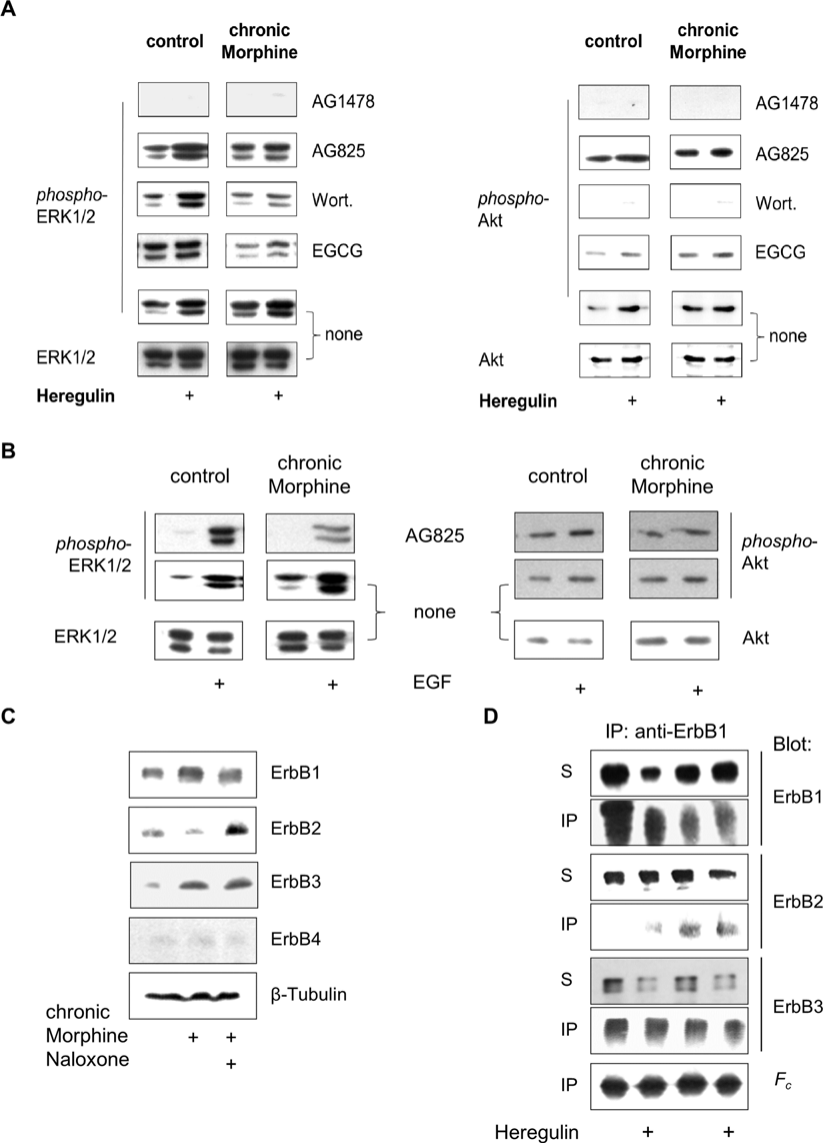
Analysis of chronic Morphine-induced changes in ErbB signalling pathways. (**A**) Effect of protein kinase blockers on basal and Heregulin-stimulated ERK1/2 and Akt phosphorylation. BT474 cells were cultured for 5 d in the absence (control) or presence of Morphine (10 μM), before the impact of ErbB1 (AG1478), ErbB2 (AG825), PI3K (Wortmannin; Wort.) and metalloproteinases (EGCG) on basal and Heregulin (40 ng/ml)-stimulated ERK1/2 and Akt signalling was determined by Western blot using phospho-specific antibodies. Controls were kept in the absence of protein inhibitors. To verify equal protein loading, controls were also stained with overall reactive anti-ERK1/2 and anti-Akt antibodies. (**B**) Involvement of ErbB2 on basal and EGF-stimulated ERK1/2 and Akt signalling. Controls and cells chronically exposed to Morphine (10 μM; 5d) were incubated with or without AG825 (50 μM; 30 min), before ERK1/2 and Akt phosphorylation was determined for 5 min in the absence or presence of EGF (100 ng/ml). Equal protein loading was verified by staining controls with overall reactive anti-ERK1/2 and anti-Akt antibodies. (**C**) Regulation of ErbB receptor abundance by chronic Morphine treatment in BT474 cells. Cells were cultured for 5 d in the presence or absence of Morphine (10 μM) and Naloxone (100 μM) as indicated and overall ErbB receptor levels were analysed by Western blot using specific antibodies for ErbB1 (175 kDa), ErbB2 (185 kDa), ErbB3 (185 kDa) and ErbB4 (170 kDa). Equal protein loading was verified by incubation of the blots with an antibody against β-tubulin. (**D**) Alteration of ErbB1 containing receptor dimers by chronic Morphine. Controls and chronically Morphine (10 μM; 5 d)-treated BT474 cells were stimulated for 5 min with or without Heregulin (40 ng/ml) to form receptor dimers. Proteins were cross-linked and ErbB1 containing dimers were immunoprecipitated using an anti-ErbB1 antibody. Individual ErbB receptors were determined in whole cell solubilisates (S) and immunoprecipitates (IP) by Western blot using type-specific antibodies. Equal protein load was verified by determination of IgG heavy chains (*F*_*c*_).

## Supporting Information

S1 File Raw Blots for Fig 4A and Fig 5A. (ZIP)
