# Supplementary figures and images for: Correction: Chronic Morphine Treatment Attenuates Cell Growth of Human BT474 Breast Cancer Cells by Rearrangement of the ErbB Signalling Network
Source: PLoS One. 2015 Oct 9;10(10):e0140727. doi: 10.1371/journal.pone.0140727 (PMC4599909; doi:10.1371/journal.pone.0140727)

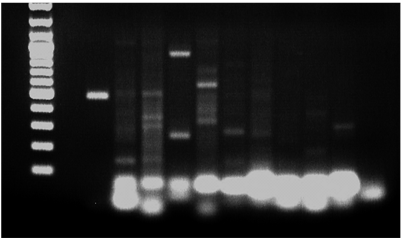

Supplement: S1 Image — (TIF) [file pone.0140727.s001.tif]

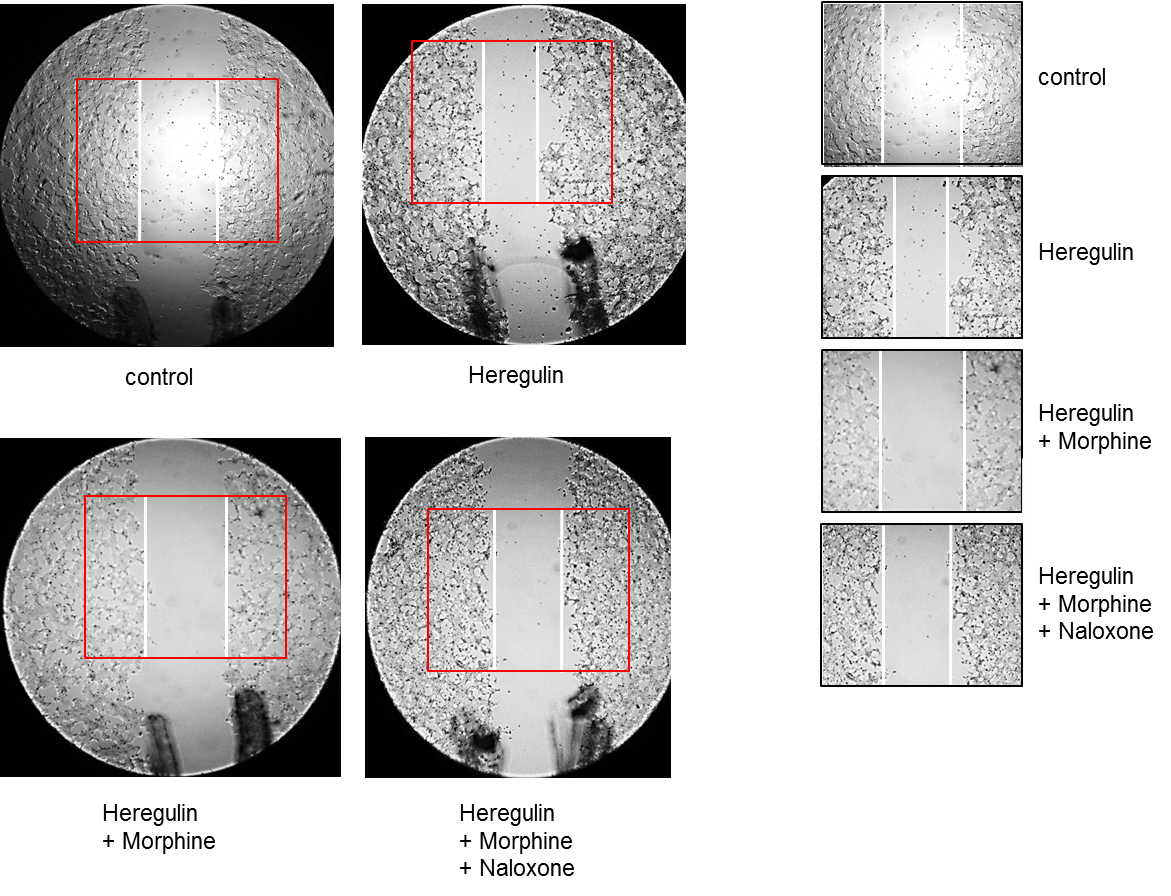

Supplement: S2 Image — (TIF) [file pone.0140727.s002.tif]
