# Supplementary material for: Correction: Chronic Morphine Treatment Attenuates Cell Growth of Human BT474 Breast Cancer Cells by Rearrangement of the ErbB Signalling Network
Source: PLoS One. 2016 Apr 14;11(4):e0153824. doi: 10.1371/journal.pone.0153824 (PMC4831685; doi:10.1371/journal.pone.0153824)
Supplement: S1 File — (ZIP) [file pone.0153824.s001.zip › Fig4 Blots.pdf]

Summary of raw blots used for construction of Figure 4 (Regulation of cell survival and apoptosis by Morphine):

A) Determination of Akt activation in control and chronically Morphine (10 µM; 5d)-treated cells.

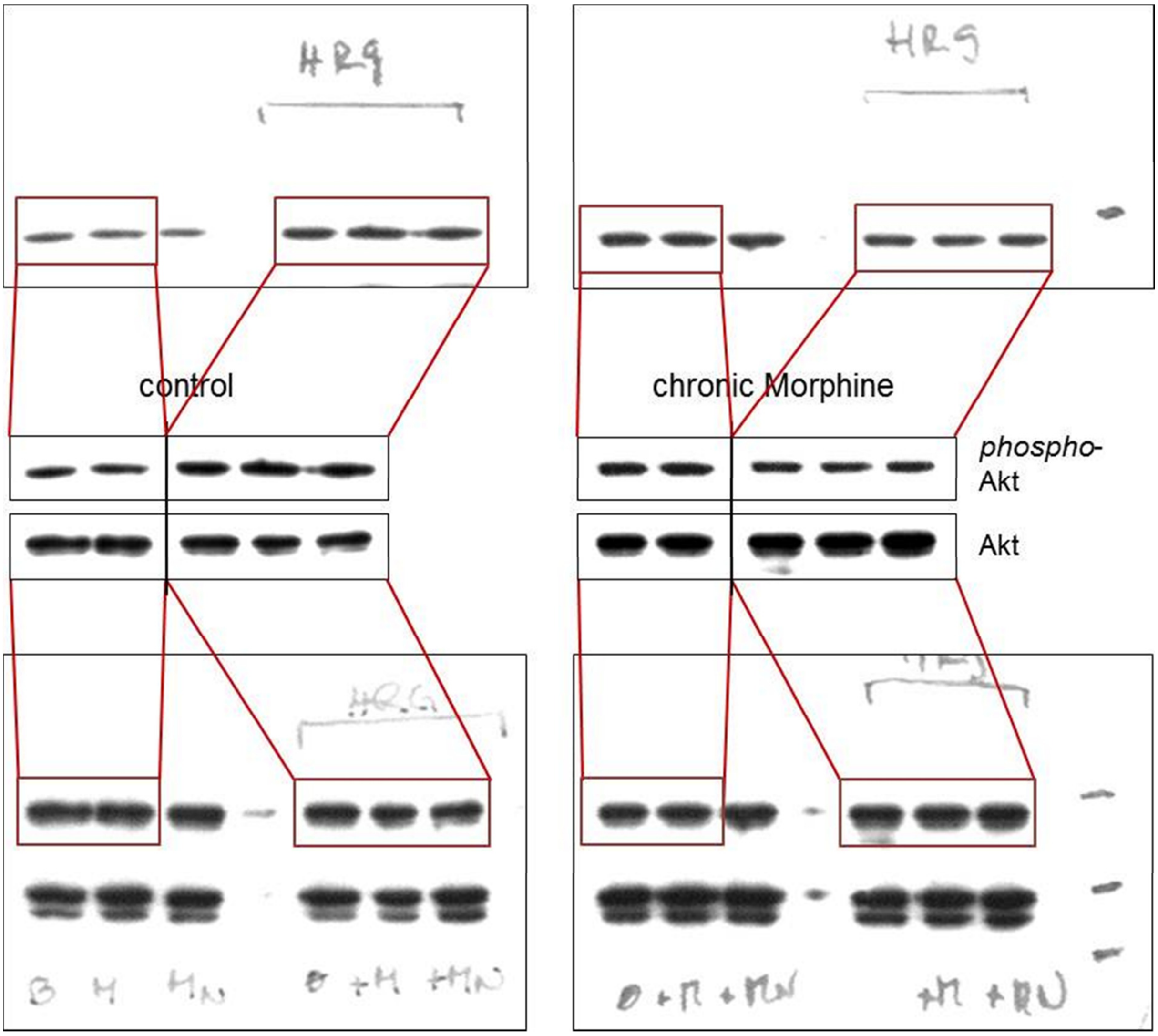

Western blots demonstrating opioid regulation of Akt phosphorylation (upper panels) in control and chronically morphine-treated BT474 cells were prepared on July 11, 2012, using electrophoresis samples from two different experiments (May 15, 2012, June 5, 2012). The blots were first stained with phospho-Akt (pS473) and phospho-ERK1/2 antibodies from Cell Signaling (cat. #9271S, #4376S). The membranes were then stripped and incubated with overall (pan)-reactive Akt and ERK1/2 antibodies also from Cell Signaling (cat. # 2920S; #9102) (done on July 25, 2012). The phospho-Akt panel of control cells is taken from the experiment done on June 5, 2012. All other Western blot panels are taken from the experiment done on May 15, 2012. The position of prestained molecular weight markers (Broad range SDS weight markers, Biorad) are indicated to the right (66 kDa, top; 66, 45 and 31 kDa bottom).

B) Comparison of basal and Heregulin (40 ng/ml)-stimulated Akt activation in control and Morphine (10 µM; 5d)-treated cells.

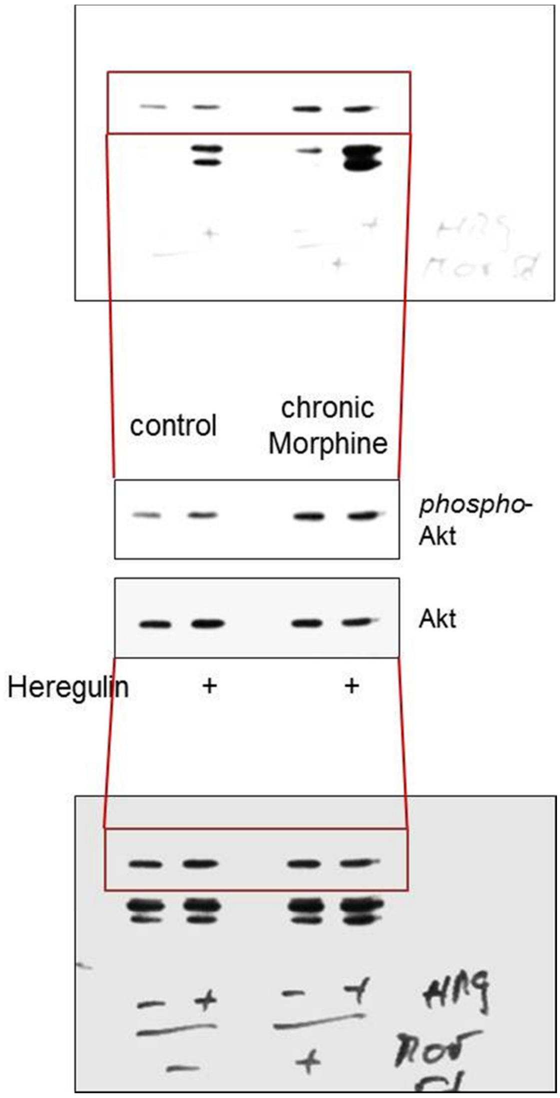

The Western blot demonstrating the difference in basal and Heregulin-ß1 stimulated Akt phosphorylation between naïve and chronically Morphine treated BT474 cells was done on November 9, 2012. The blot was first developed with pan-Akt and pan-ERK1/2 antibodies, stripped and then incubated with phospho-Akt (pS473) and phospho-ERK1/2 antibodies on November 13, 2012.

C) Determination of PARP cleavage in BT474 cells.

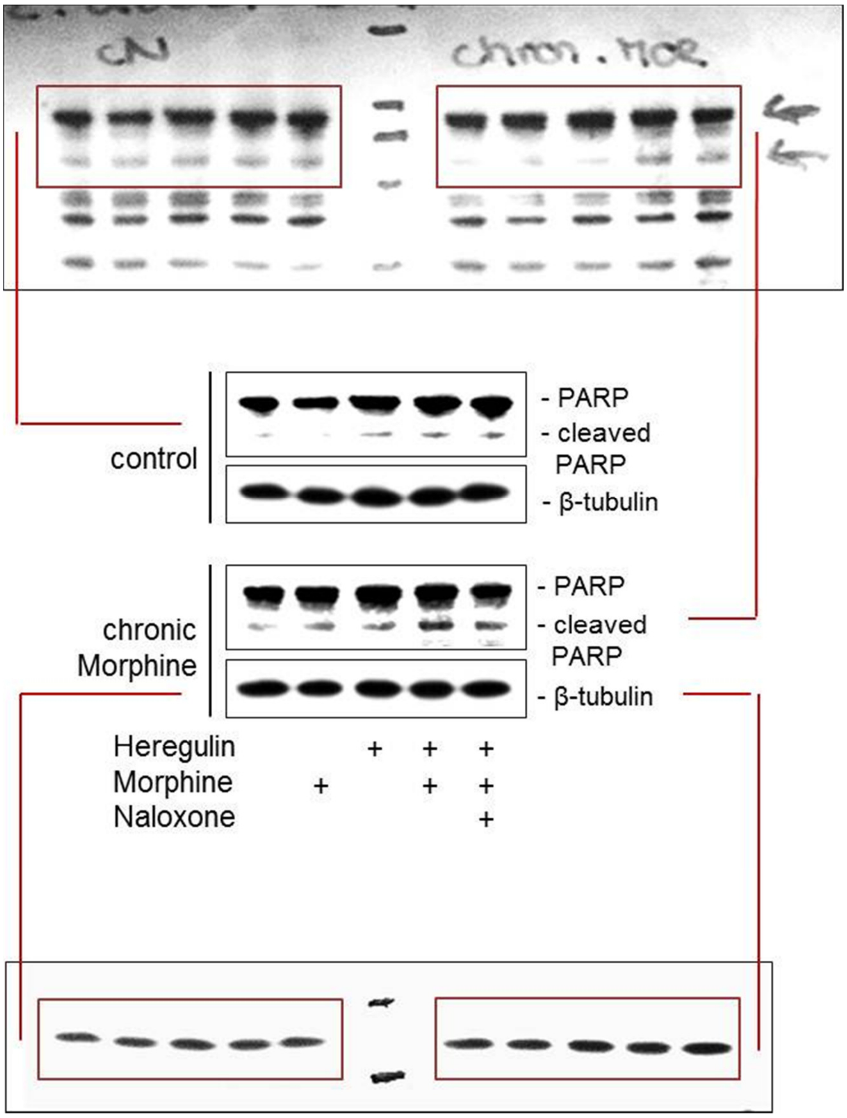

The Western blots demonstrating PARP cleavage by Morphine and Heregulin-ß1 in naïve and chronically Morphine treated BT474 cells were done on August 16, 2012. The blots were either stained with antibodies that recognize total PARP (Cell Signaling, cat. # 9542; 116 kDa = uncleaved; 89 kDa = cleaved at Asp214; top) or ß-tubulin (Cell Signaling, cat. # 2128; 55 kDa; bottom). The position of prestained molecular weight markers (Broad range SDS weight markers, BioRad) are indicated between the two sets of samples on each gel (200, 116, 97, 66 kDa, top gel; 66 and 45 kDa bottom gel).
