# Supplementary material for: Correction: Chronic Morphine Treatment Attenuates Cell Growth of Human BT474 Breast Cancer Cells by Rearrangement of the ErbB Signalling Network
Source: PLoS One. 2016 Apr 14;11(4):e0153824. doi: 10.1371/journal.pone.0153824 (PMC4831685; doi:10.1371/journal.pone.0153824)
Supplement: S1 File — (ZIP) [file pone.0153824.s001.zip › pone.0153824.docx]

# Correction: Chronic Morphine Treatment Attenuates Cell Growth of Human BT474 Breast Cancer Cells by Rearrangement of the ErbB Signalling Network

**Inka Regine Weingaertner, Sarah Koutnik, Hermann Ammer**

There are undisclosed splices between lanes 2 and 3 for each panel in Figure 4A. Because no acute opioid effect on Akt phosphorylation was observed in naïve cells, the authors spliced out the Naloxone control band. The authors have provided a corrected version of Fig 4, in which all splices are clearly demarcated with a vertical black line. The raw blots for each panel in Fig 4A are provided below as supporting information.

Additionally, there is an error in Figure 5A that was introduced during figure preparation. In the right half of Figure 5A, the lowest control panel is erroneously a duplicate of the first two lanes of the lower control panel in Figure 4A. The authors have provided a correct version of Fig 5. The raw blots for Fig 5A are provided below as supporting information.

## Supporting Information

**S1 File.** **Raw Blots for Fig 4A and Fig 5A.**

## Reference

1. Weingaertner IR, Koutnik S, Ammer H (2013) Chronic Morphine Treatment Attenuates Cell Growth of Human BT474 Breast Cancer Cells by Rearrangement of the ErbB Signalling Network. PLoS ONE 8(1): e53510. doi:10.1371/journal.pone.0053510
