# Supplementary figures and images for: Correction: Chronic Morphine Treatment Attenuates Cell Growth of Human BT474 Breast Cancer Cells by Rearrangement of the ErbB Signalling Network
Source: PLoS One. 2016 Apr 14;11(4):e0153824. doi: 10.1371/journal.pone.0153824 (PMC4831685; doi:10.1371/journal.pone.0153824)

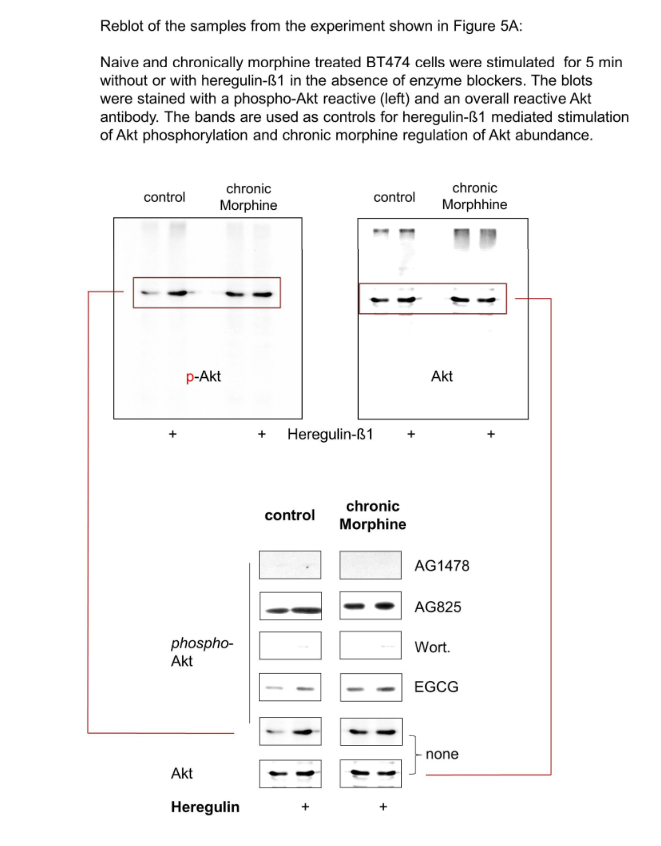

Supplement: S1 File — (ZIP) [file pone.0153824.s001.zip › Fig5A Blots.tiff]

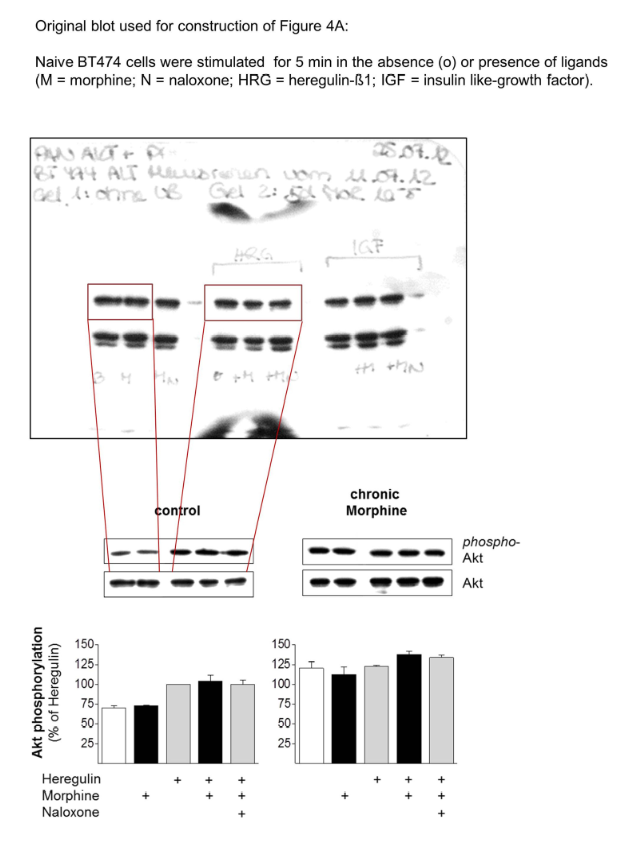

Supplement: S1 File — (ZIP) [file pone.0153824.s001.zip › Fig4A Blots.tiff]
